# Prognostic and predictive effects of new steatotic liver disease nomenclatures: a large population‐based study

**DOI:** 10.1002/mco2.70087

**Published:** 2025-02-13

**Authors:** Huixian Zeng, Letian Fang, Zhiyu Yang, Xinyu Zhao, Hongsen Chen, Puyi Xing, Zheyun Niu, Zheng Li, Zishuai Li, Jiayi Zhao, Wenbin Liu, Chunxia Jing, Hong You, Guangwen Cao

**Affiliations:** ^1^ Department of Epidemiology School of Medicine Jinan University Guangzhou Guangdong China; ^2^ Key Laboratory of Biological Defense Ministry of Education Second Military Medical University Shanghai China; ^3^ Shanghai Key Laboratory of Medical Bioprotection Second Military Medical University Shanghai China; ^4^ Department of Epidemiology Second Military Medical University Shanghai China; ^5^ Jiading District Center for Disease Control and Prevention Shanghai China; ^6^ Department of Vitral Statistics Shanghai Municipal Center for Disease Control and Prevention Shanghai China; ^7^ Clinical Epidemiology & EBM Unit Beijing Friendship Hospital Capital Medical University National Clinical Research Center for Digestive Diseases Beijing China; ^8^ Shanghai East Hospital Key Laboratory of Arrhythmias Ministry of Education Tongji University School of Medicine Tongji University Shanghai China; ^9^ Liver Research Center Beijing Friendship Hospital Capital Medical University State Key Lab of Digestive Health National Clinical Research Center of Digestive Diseases Beijing China

**Keywords:** antiglycemic medication, diabetes, metabolic dysfunction‐associated fatty liver disease, metabolic dysfunction‐associated steatotic liver disease, prognosis

## Abstract

We aimed to compare the association of metabolic dysfunction‐associated fatty liver disease (MAFLD), metabolic dysfunction‐associated steatotic liver disease (MASLD), alcohol‐related liver disease (ALD), metabolic dysfunction and ALD (MetALD), and MASLD with viral hepatitis (MASLD‐Viral) with risks of cirrhosis, liver cancer, and mortality. The data of 464,556 adults from the UK Biobank (UKB), 13,526 adults from the National Health and Nutrition Examination Survey (NHANES), and 2554 adults from BeijngFH Health Cohort Study (FHCS) were included. Adjusted hazard ratios (aHR) and odds ratios were calculated using Cox and Logistic regression models, respectively. Compared with non‐SLD, the risk of liver cancer increased from MetALD (aHR 1.70 [95% CI 1.37, 2.09]), MASLD (1.91 [1.66, 2.21]), MAFLD (2.01 [1.76, 2.29]), ALD (3.16 [2.54, 3.93]), to MASLD‐Viral (22.0 [10.8, 44.4]) in a stepwise manner in the UKB; the risk of all‐cause mortality increased from MetALD, MASLD, MAFLD, ALD, to MASLD‐Viral in the NHANES. The odds ratio of liver fibrosis increased from MASLD, MAFLD, to MASLD‐Viral in the FHCS. In patients with diabetes, metformin plus other drugs were associated with higher risks of cirrhosis, liver cancer, and all‐cause mortality in MASLD or MAFLD. Prevention rather than antiglycemic treatment is important for patients with diabetic MASLD or MAFLD.

## Graphical abstract

This study analyzed the UK Biobank, NHANES III, and FHCS data to assess the prognostic and associative effects of new steatotic liver disease nomenclatures. It found that MAFLD, MASLD, and MetALD were associated with higher risks of liver‐related conditions and mortality, with diabetes and certain antiglycemic medications further heightening these risks

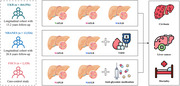

## 1 INTRODUCTION

Nonalcoholic fatty liver disease (NAFLD), one of the leading causes of liver cirrhosis and liver cancer, has a global prevalence of 25%.^1^, ^2^ Since the proposal of the term NAFLD, which is characterized by hepatic steatosis in the absence of excessive alcohol consumption and other causes of fatty liver, there has been considerable debate concerning its drawbacks, namely, the exclusionary nature of the term “nonalcoholic” and stigma associated with the use of the term “fatty.”^3^ The redefined term, metabolic dysfunction‐associated fatty liver disease (MAFLD), encompasses hepatic steatosis alongside the presence of overweight, type 2 diabetes, and/or a combination of two out of seven metabolic risk factors, including central obesity, hypertension, prediabetes, hypertriglyceridemia, low plasma high‐density lipoprotein (HDL), elevated high‐sensitive C‐reactive protein, and insulin resistance.^4^, ^5^ However, MAFLD was also criticized for relying solely on metabolic risk factors, neglecting alcohol consumption and nonalcoholic steatohepatitis.^3^ In 2023, metabolic dysfunction‐associated steatotic liver disease (MASLD) was proposed to replace NAFLD and MAFLD, and steatotic liver disease (SLD) was suggested as an umbrella term. MASLD was defined by the presence of SLD with one or more of five cardiometabolic risk factors (central obesity, hyperglycemia, hypertension, hypertriglyceridemia, and low HDL) alongside self‐reported alcohol intake below 20 g/day for women or 30 g/day for men.^3^, ^6^ Additionally, new subcategories, namely metabolic dysfunction and alcohol‐related steatotic liver disease (MetALD), alcohol‐related liver disease (ALD), and MASLD‐viral hepatitis (MASLD‐Viral), have been proposed to classify patients with MASLD based on their alcohol consumption levels and presence of viral hepatitis. This updated terminology introduces a positive diagnostic criterion, integrates metabolic risk factors, and underscores the importance of addressing manageable risk factors such as alcohol consumption and viral hepatitis. However, the impact of the new nomenclature on patient outcomes remains uncertain.

Several studies have demonstrated a similar prevalence of NAFLD, MAFLD, and MASLD among different populations,^7^, ^8^, ^9^, ^10^ with MAFLD exhibiting a slightly higher prevalence.^9^ However, their prognostic values vary across studies. Compared with NAFLD, MAFLD and MASLD are associated with an increased risk of all‐cause mortality.^11^, ^12^ In addition, MAFLD and NAFLD have similar clinical profiles and long‐term outcomes.^13^ Another study has demonstrated that the MAFLD‐only and MASLD/MAFLD overlap subgroups have higher all‐cause mortality rates than the MASLD‐only subgroup.^14^ The disparities lies in the different criteria for metabolic factors, alcohol intake, and viral hepatitis.^14^, ^15^ Thus, prospective cohort studies are needed to address the prognosis of patients with MAFLD and MASLD.

We conducted a comprehensive study using data from two cohorts, the US National Health and Nutrition Examination Survey (NHANES) and UK Biobank (UKB), to explore the association of MAFLD and SLD subclassifications with liver‐related adverse outcomes and validated it with a case–control study from BeijingFH Health Cohort Study (FHCS). We sought to determine whether the novel nomenclature effectively stratifies patients with SLD based on their propensity to develop liver disease and lead to death, thus requiring specific interventions. We also evaluated the impact of diabetes, an important risk factor, on the prognosis of patients with MAFLD or MASLD and attempted to uncover the role of antiglycemic medications in the diabetic steatotic liver population.

## 2 RESULTS

### 2.1 Baseline characteristics

**FIGURE 1:**
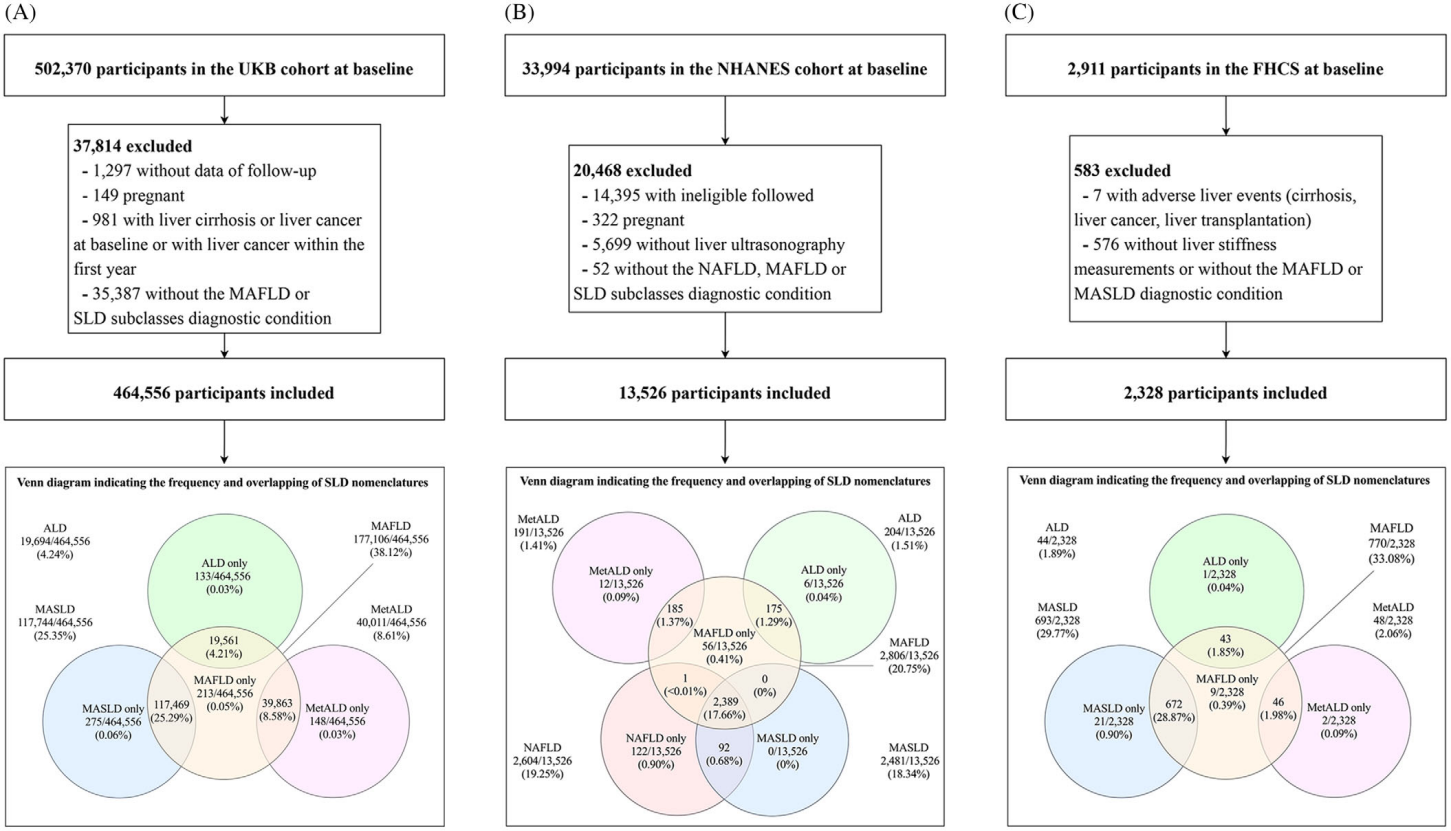
The flow chart and Venn diagram of SLD nomenclatures in the three studies. (A) The UKB cohort. (B) The NHANES cohort. (C) The FHCS study. NAFLD, nonalcoholic fatty liver disease; SLD, steatotic liver diseases; MAFLD, metabolic dysfunction‐associated fatty liver disease; MASLD, metabolic dysfunction‐associated steatotic liver disease; MetALD, MASLD with increased alcohol intake; ALD, alcohol‐related liver disease; UKB, UK Biobank; NHANES, US National Health and Nutrition Examination Survey; FHCS, BeijingFH Health Cohort Study.

In the UKB cohort, participants lost to follow‐up (*n* = 1297), pregnant women (*n* = 149), those with liver cirrhosis or liver cancer at baseline or with liver cancer within the first year (*n* = 981), or those without the diagnostic condition of MAFLD or SLD subclasses (*n* = 35,387) were excluded. Finally, 464,556 adults were included (Figure 1A). The mean participant age was 56.5 years, 212,370 (45.7%) were men, and 439,823 (94.7%) were white individuals. The prevalence rates of diabetes and cardiovascular diseases (CVDs) were 6.9% (*n* = 28,445) and 6.7% (*n* = 31,320), respectively (Table 1). In total, 177,106 (38.1%), 117,744 (25.3%), 40,011 (8.6%), 19,694 (4.2%), and 214 (<0.1%) participants met the diagnostic criteria for MAFLD, MASLD, MetALD, ALD, and MASLD‐Viral, respectively (Table 1). NAFLD was not identified in the UKB cohort. Baseline characteristics of the MAFLD and MASLD groups are presented in Tables S1–S2. In the NHANES cohort, 13,526 adults were included in the analysis after excluding individuals who were not eligible for follow‐up (*n* = 14,395); pregnant women (*n* = 322); those without liver ultrasonography records (*n* = 5699); and those without diagnoses for the subcategories of NAFLD, MAFLD, or SLD (*n* = 52) (Figure 1B). The mean participant age was 43.9 years, 6442 (47.6%) were men, and 8929 (66.0%) were white individuals. The prevalence of diabetes was 13.3% (*n* = 1784), and 2604 (19.3%), 2806 (20.7%), 2481 (18.3%), 191 (1.4%), 204 (1.5%), and 62 (0.5%) participants met the diagnostic criteria for NAFLD, MAFLD, MASLD, MetALD, ALD, and MASLD‐Viral, respectively. Notably, 2387 (17.7%) participants met the criteria for NAFLD, MAFLD, and MASLD. In the FHCS, 2554 participants were included after excluding 7 with liver cirrhosis, liver cancer, or liver transplantation and 576 without FibroScan results and without the MAFLD or MASLD diagnostic condition (Figure 1C). The prevalence rates of MAFLD, MASLD, MetALD, ALD, and MASLD‐Viral were 33.1% (*n* = 770), 29.8% (*n* = 693), 2.1% (*n* = 48), 1.9% (*n* = 44), and 0.4% (*n* = 10), respectively. The mean participant age was 41.4 years, and 1472 (63.2%) were men. Baseline characteristics of participants in the FHCS are detailed in Table 1.

**TABLE 1:** The baseline characteristics and follow‐up summaries of all participants in the UKB and NHANES cohorts.

	UKB (*N* = 464,556)	NHANES (*N* = 13,526)	FHCS (*N* = 2,328)
Male, *n* (%)	212,370 (45.7)	6442 (47.6)	1472 (6.2)
Age, mean (SD) (years)	56.5 (8.1)	43.9 (16.0)	41.4 (3.4)
Race, *n* (%)			
White	439,823 (94.7)	8,929 (66.0)	0.0 (0.0)
Other	24,733 (5.3)	4597 (34.0)	2328 (100.0)
Alcohol intake, *n* (%)			
Mild	326,799 (70.3)	11,911 (88.1)	2165 (93.0)
Moderate	103,611 (22.3)	817 (6.0)	89 (3.8)
Severe	34,146 (7.4)	798 (5.9)	74 (3.2)
Cigarette smoking, *n* (%)			
Never smoker	254,494 (54.8)	6528 (48.3)	1862 (80.0)
Past smoker	161,268 (34.7)	3144 (23.2)	215 (9.2)
Current smoker	48,794 (10.5)	3854 (28.5)	251 (10.8)
Waist circumference, mean (SD) (cm)	90.3 (13.4)	93.0 (14.7)	84.8 (12.1)
BMI, mean (SD) (kg/m2)	27.4 (4.8)	27.3 (5.9)	24.4 (3.4)
SBP, mean (SD) (mmHg)	139.7 (19.7)	122.0 (19.3)	116.7 (14.6)
DBP, mean (SD) (mmHg)	82.2 (10.7)	73.4 (11.8)	70.5 (10.3)
Hypertension, *n* (%)	337,379 (72.6)	5585 (41.3)	362 (15.5)
Cardiovascular disease, *n* (%)	31,320 (6.7)	812 (6.0)	38 (1.6)
Diabetes stage, *n* (%)			
Without diabetes	325,003 (79.0)	7405 (55.4)	2202 (94.6)
Prediabetes	58,149 (14.1)	4188 (31.3)	–
Diabetes	28,445 (6.9)	1784 (13.3)	126 (5.4)
History of cancer, *n* (%)	46,886 (10.1)	709 (5.2)	74 (3.2)
Viral hepatitis, *n* (%)	694 (0.1)	379 (2.8)	33 (1.4)
Fib‐4, median (IQR)	0.9 (0.7, 1.3)	0.8 (0.5, 1.2)	0.9 (0.7, 1.1)
Glycated hemoglobin, median (IQR) (%)	5.4 (5.2, 5.6)	5.3 (5.0, 5.7)	5.4 (5.2, 5.7)
Total cholesterol, mean (SD) (mg/dL)	220.1 (44.2)	204.2 (44.2)	190.0 (33.5)
HDL‐cholesterol, mean (SD) (mg/dL)	56.0 (14.8)	50.8 (15.4)	51.3 (12.0)
Triglycerides, median (IQR) (mg/dL)	131.3 (92.6, 190.2)	112.0 (78.0, 170.0)	99.2 (68.2, 148.8)
AST, median (IQR) (U/L)	20.1 (15.4, 27.4)	19.0 (16.0, 24.0)	21.5 (18.4, 26.2)
ALT, median (IQR) (U/L)	24.4 (21.0, 28.8)	14.0 (10.0, 21.0)	19.0 (13.0, 28.0)
GGT, median (IQR) (U/L)	26.3 (18.5, 40.8)	22.0 (15.0, 36.0)	–
CRP, median (IQR) (mg/L)	1.3 (0.6, 2.8)	2.1 (2.1, 4.0)	–
HOMA‐IR, median (IQR)	–	2.1 (1.4, 3.4)	–
NAFLD, *n* (%)	–	2604 (19.3)	–
MAFLD, *n* (%)	177,106 (38.1)	2806 (20.7)	770 (33.1)
SLD, *n* (%)			
MASLD, *n* (%)	117,744 (25.3)	2481 (18.3)	693 (29.8)
MetALD, *n* (%)	40,011 (8.6)	191 (1.4)	48 (2.1)
ALD, *n* (%)	19,694 (4.2)	204 (1.5)	44 (1.9)
MASLD‐Viral, *n* (%)	214 (<0.1)	62 (0.5)	10 (0.4)
Follow‐up time, median (IQR) (years)	13.2 (12.5, 14.0)	26.8 (21.2, 28.8)	–
Outcomes			
No. of liver cirrhosis, *n* (%)	2458 (0.5)	–	–
No. of liver cancer, *n* (%)	1019 (0.2)	–	–
No. of deaths, cancer, *n* (%)	18,345 (3.9)	1170 (8.7)	–
No. of deaths, all‐causes, *n* (%)	37,625 (8.1)	4858 (35.9)	–

Continuous data are presented as mean (standard deviation) if normally distributed or as median (interquartile range) if non‐normally distributed. Categorical data are expressed as number (%).

Abbreviations: BMI, body mass index; SBP, systolic blood pressure; DBP, diastolic blood pressure; Fib‐4, fibrosis‐4 index; HDL, high‐density lipoprotein; AST, aspartate aminotransferase; ALT, alanine aminotransferase; GGT, gamma‐glutamyl transpeptidase; CRP, C‐reactive protein; HOMA‐IR, homeostatic model assessment of Insulin resistance; NAFLD, nonalcoholic fatty liver; MAFLD, metabolic dysfunction‐associated fatty liver disease; MASLD, metabolic dysfunction‐associated steatotic liver disease; MetALD, MASLD with increased alcohol intake; MASLD‐Viral, MASLD with chronic viral hepatitis; ALD, alcohol‐related liver disease; SD, standard deviation; IQR, interquartile range; UKB, UK Biobank; NHANES, National Health and Nutrition Examination Survey; FHCS, BeijingFH Health Cohort Study.

The Venn diagrams depicted in Figure 1 illustrate the intersection of SLD nomenclatures across the three studies. The definition of MAFLD is broader than that of MASLD and encompasses other disorders such as ALD and MetALD. In the UKB, the proportion of MAFLD (38.12%) was similar to the sum of the proportions of MASLD (25.35%), ALD (4.24%), and MetALD (8.61%). These findings are consistent across both the NHANES and FHCS. Notably, in the NHANES, all participants with MASLD met the diagnostic criteria for NAFLD. The proportions of SLD subtypes in the UKB and NHANES are shown in Figure S1.

**FIGURE 2:**
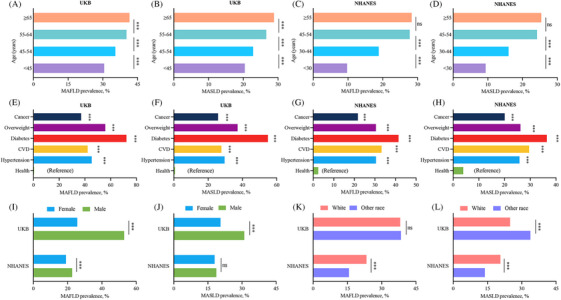
The prevalence of MAFLD and MASLD in participants stratified by age, sex, race, and chronic conditions in the UKB and NHANES cohorts. Prevalence of MAFLD in the UKB participants stratified by age. (B) Prevalence of MASLD in the UKB participants stratified by age. (C) Prevalence of MAFLD in the NHANES participants stratified by age. (D) Prevalence of MASLD in the NAHNES participants stratified by age. (E) Prevalence of MAFLD in the UKB participants stratified by chronic conditions. (F) Prevalence of MASLD in the UKB participants stratified by chronic conditions. (G) Prevalence of MAFLD in the NHANES participants stratified by chronic conditions. (H) Prevalence of MASLD in the NAHNES participants stratified by chronic conditions. (I) Prevalence of MAFLD in the UKB and NHANES participants stratified by sex. (J) Prevalence of MASLD in the UKB and NHNAES participants stratified by sex. (K) Prevalence of MAFLD in the UKB and NHANES participants stratified by race. (L) Prevalence of MASLD in the UKB and NAHNES participants stratified by race. All bars in the figure indicate the Prevalence of MAFLD or MASLD, and comparisons of frequencies between groups were made using the Wilcoxon test or chi‐square test. Different age groups or different disease states are distinguished by different colors. Overweight in E, F, G, and H refers to participants who are overweight or obese. Healthy participants are those without cancer, cardiovascular disease, diabetes, hypertension, and overweight or obesity at baseline. MAFLD, metabolic dysfunction‐associated fatty liver disease; MASLD, metabolic dysfunction‐associated steatotic liver disease; UKB, UK Biobank; NHANES, National Health and Nutrition Examination Survey. * Denotes *p *< 0.05, ** denotes *p *< 0.01, *** denotes *p *< 0.001, and “ns” denotes lack of statistical significance of the observed differences.

Figure 2 shows the prevalence of MAFLD and MASLD in participants stratified by age, sex, race, and chronic conditions in the UKB and NHANES cohorts. The prevalence of MAFLD and MASLD increased with age and were higher in participants with chronic conditions (hypertension, CVD, diabetes, overweight/obesity, and cancer) than in healthy controls in both cohorts. In the UKB, the prevalence of MAFLD and MASLD were higher in male participants than in female participants. In the NHANES, MAFLD was more prevalent among male participants compared with female participants, but no significant sex‐based differences were noted for MASLD. Interestingly, in the NHANES cohort, a higher prevalence of both MAFLD and MASLD was observed among white individuals, whereas in the UKB data, MASLD was less prevalent among white individuals and no statistical difference was observed for the prevalence of MAFLD. These observations highlight the importance of considering demographic factors when examining the prevalence of metabolism‐associated liver conditions. Overall, the findings of the UKB are largely consistent with those of the NHANES, indicating a broad applicability of these results across different populations.

### 2.2 Association of SLD nomenclatures with adverse liver outcomes

We evaluated the association between the four new nomenclatures and the risk of developing liver cirrhosis and cancer in the UKB cohort (Table 2). During follow‐up (median 13.2 years), 2458 participants had newly developed liver cirrhosis, 1019 had newly developed liver cancer, 37,625 died, and 18,345 died of cancer (Table 1). The models were adjusted for age, sex, race, smoking status, CVD, cancer history, and fibrosis‐4 index (Fib‐4) score, with additional adjustments for alcohol intake and viral hepatitis in MAFLD. Compared with non‐SLD, MAFLD, MASLD, MetALD, ALD, and MASLD‐Viral were simultaneously and independently associated with an increased risk of cirrhosis, with an adjusted hazard ratio (aHR) (95% confidence interval [CI]) of 3.37 (3.08, 3.70), 3.04 (2.75, 3.36), 3.00 (2.62, 3.43), 7.38 (6.49, 8.40), and 32.10 (20.20, 50.80), respectively. Furthermore, MAFLD, MASLD, MetALD, ALD, and MASLD‐Viral were concurrently and independently associated with an increased risk of liver cancer, with an aHR (95% CI) of 2.01 (1.76, 2.29), 1.91 (1.66, 2.21), 1.70 (1.37, 2.09), 3.16 (2.54, 3.93), and 22.00 (10.80, 44.40), respectively. Thus, individuals with a higher risk of cirrhosis and liver cancer could be distinguished using the MAFLD criteria compared with the MASLD criteria. Compared with non‐SLD, MAFLD, MASLD, MetALD, ALD, and MASLD‐Viral were independently and simultaneously associated with an increased risk of cancer‐related mortality, with an aHR (95% CI) of 1.29 (1.25, 1.33), 1.32 (1.28, 1.36), 1.18 (1.12, 1.24), 1.38 (1.29, 1.47), and 1.81 (1.07, 3.06), respectively. In addition, MAFLD, MASLD, MetALD, ALD and MASLD‐Viral were independently and simultaneously associated with an increased risk of all‐cause mortality, with an aHR (95% CI) of 1.34 (1.31, 1.37), 1.39 (1.36, 1.42), 1.18 (1.14, 1.22), 1.44 (1.38, 1.51) and 1.90 (1.35, 2.68), respectively (Table 2). Unsurprisingly, the risks of all‐cause and cancer‐related mortality in participants with MASLD‐Viral were the highest among the four nomenclature groups.

**TABLE 2:** Association of MAFLD and subtypes of steatotic liver disease with cirrhosis, liver cancer, cancer‐related mortality, and all‐cause mortality.

	Total	Case	Rate^a^	Unadjusted HR (95% CI)	Adjusted HR (95% CI)	*p* Value
**UKB (*N* = 464,556)**						
**Cirrhosis**						
Without hepatic steatosis	286,871	701	0.19	1.00 (Reference)	1.00 (Reference)	
MAFLD^b^	177,106	1734	0.76	4.09 (3.75, 4.47)	3.37 (3.08, 3.70)	<0.001
SLD^c^						
MASLD	117,744	953	0.63	3.39 (3.08, 3.74)	3.04 (2.75, 3.36)	<0.001
MetALD	40,011	343	0.67	3.55 (3.12, 4.04)	3.00 (2.62, 3.43)	<0.001
ALD	19,694	440	7.51	9.44 (8.38, 10.6)	7.38 (6.49, 8.40)	<0.001
MASLD‐Viral	214	19	1.76	41.5 (26.3, 65.4)	32.1 (20.2, 50.8)	<0.001
**Liver cancer**						
Without hepatic steatosis	286,871	406	0.11	1.00 (Reference)	1.00 (Reference)	
MAFLD^b^	177,106	612	0.27	2.49 (2.20, 2.82)	2.01 (1.76, 2.29)	<0.001
SLD^c^						
MASLD	117,744	368	0.24	2.25 (1.96, 2.60)	1.91 (1.66, 2.21)	<0.001
MetALD	40,011	121	0.23	2.16 (1.76, 2.65)	1.70 (1.37, 2.09)	<0.001
ALD	19,694	116	3.08	4.28 (3.48, 5.26)	3.16 (2.54, 3.93)	<0.001
MASLD‐Viral	214	8	0.46	29.0 (14.4, 58.3)	22.0 (10.8, 44.4)	<0.001
**Cancer‐related mortality**						
Without hepatic steatosis	286,871	9593	2.57	1.00 (Reference)	1.00 (Reference)	
MAFLD^b^	177,106	8728	3.84	1.50 (1.46, 1.54)	1.29 (1.25, 1.33)	<0.001
SLD^c^						
MASLD	117,744	5712	3.78	1.48 (1.43, 1.53)	1.32 (1.28, 1.36)	<0.001
MetALD	40,011	1895	3.67	1.43 (1.36, 1.50)	1.18 (1.12, 1.24)	<0.001
ALD	19,694	1129	5.32	1.76 (1.65, 1.87)	1.38 (1.29, 1.47)	<0.001
MASLD‐Viral	214	14	4.50	2.11 (1.25, 3.56)	1.81 (1.07, 3.06)	<0.001
**All‐cause mortality**						
Without hepatic steatosis	286,871	18,516	4.96	1.00 (Reference)	1.00 (Reference)	
MAFLD^b^	177,106	19,040	8.38	1.70 (1.66, 1.73)	1.34 (1.31, 1.37)	<0.001
SLD^c^						
MASLD	117,744	12,573	8.33	1.69 (1.65, 1.73)	1.39 (1.36, 1.42)	<0.001
MetALD	40,011	3971	7.69	1.55 (1.50, 1.61)	1.18 (1.14, 1.22)	<0.001
ALD	19,694	2529	12.55	2.04 (1.96, 2.13)	1.44 (1.38, 1.51)	<0.001
MASLD‐Viral	214	33	10.08	2.60 (1.85, 3.66)	1.90 (1.35, 2.68)	<0.001
**NHANES (*N* = 13,526)**						
**Cancer‐related mortality**						
Without hepatic steatosis	10,465	856	3.39	1.00 (Reference)	1.00 (Reference)	
NAFLD^c^	2604	258	4.40	1.32 (1.15, 1.52)	1.27 (1.10, 1.46)	<0.001
MAFLD^b^			4.73	1.42 (1.25, 1.63)	1.31 (1.14, 1.50)	<0.001
SLD^c^						
MASLD	2481	256	4.62	1.39 (1.21, 1.60)	1.31 (1.13, 1.51)	<0.001
MetALD	191	22	5.31	1.60 (1.05, 2.45)	1.09 (0.70, 1.71)	0.698
ALD	204	22	5.18	1.57 (1.03, 2.39)	1.44 (0.94, 2.20)	0.093
MASLD‐Viral	62	12	10.04	3.07 (1.74, 5.44)	3.07 (1.73, 5.43)	<0.001
**All‐cause mortality**						
Without hepatic steatosis	10,465	3436	13.62	1.00 (Reference)	1.00 (Reference)	
NAFLD^c^	2604	1180	20.13	1.52 (1.42, 1.62)	1.36 (1.28, 1.46)	<0.001
MAFLD^b^	2806	1069	22.00	1.66 (1.56, 1.77)	1.43 (1.34, 1.53)	<0.001
SLD^c^						
MASLD	2481	1165	21.04	1.59 (1.49, 1.70)	1.38 (1.29, 1.47)	<0.001
MetALD	191	98	23.64	1.80 (1.47, 2.20)	1.27 (1.02, 1.57)	0.032
ALD	204	107	25.20	1.92 (1.58, 2.32)	1.79 (1.48, 2.17)	<0.001
MASLD‐Viral	62	37	30.97	2.39 (1.73, 3.31)	2.62 (1.89, 3.62)	<0.001

Results were obtained with Cox proportional hazards.

Abbreviations: aHR, adjusted hazard ratio; CI, confidence interval; CVD, cardiovascular disease; Fib‐4, Fibrosis‐4 index; NAFLD, nonalcoholic fatty liver; MAFLD, metabolic dysfunction‐associated fatty liver disease; SLD, steatotic liver disease; MASLD, metabolic dysfunction‐associated steatotic liver disease; MetALD, MASLD with increased alcohol intake; ALD, alcohol‐related liver disease; MASLD‐Viral, MASLD with chronic viral hepatitis; UKB, UK Biobank; NHANES, National Health and Nutrition Examination Survey.

^a^ Incidence rate per 1000 person years.

^b^ Models were adjusted for age (category), sex, race, cigarette smoking, alcohol intake, Fib‐4, CVD, history of cancer, and viral hepatitis.

^c^ Models were adjusted for age (category), sex, race, cigarette smoking, CVD, history of cancer, and Fib‐4.

In the NHANES cohort, the definitions of cirrhosis and liver cancer were not available; therefore, the risk of all‐cause and cancer‐related mortality in participants with different SLD criteria in the NHANES cohort was evaluated. During follow‐up (median, 26.8 years), 4858 participants died, 1170 of whom died of cancer (Table 1). Compared with non‐SLD, NAFLD, MAFLD, MASLD, MetALD, ALD, and MASLD‐Viral were simultaneously and independently associated with an increased risk of all‐cause mortality, with an aHR (95% CI) of 1.36 (1.28, 1.46), 1.43 (1.34, 1.53), 1.38 (1.29, 1.47), 1.27 (1.02, 1.57), 1.79 (1.48, 2.17), and 2.62 (1.89, 3.62), respectively. Additionally, NAFLD, MAFLD, MASLD, ALD, and MASLD‐Viral were independently and simultaneously associated with an increased risk of cancer‐related mortality, with an aHR (95% CI) of 1.27 (1.10, 1.46), 1.31 (1.14, 1.50), 1.31 (1.13, 1.51), 1.09 (0.70, 1.71), 1.44 (0.94, 2.20), and 3.07 (1.73, 5.43), respectively; however, MetALD and ALD were not associated with cancer‐related mortality after adjustment (Table 2). Generally, the risks of all‐cause and cancer‐related mortality increased in a stepwise manner for MetALD, NAFLD, MASLD, MAFLD, ALD, and MASLD‐Viral. In alignment with the findings of the UKB, in the NHANES, a greater number of participants with higher risks of all‐cause and cancer‐related mortalities were identified using the MAFLD criteria compared with the MASLD criteria. The prognosis of MASLD is more similar to that of NAFLD than that of MAFLD. Furthermore, adults with severe alcohol intake and concomitant viral hepatitis who had a higher risk of mortality, especially those with concomitant viral hepatitis, were identified using the ALD and MASLD‐Viral criteria, respectively.

In the FHCS cohort, logistic regression analysis was performed to assess the association between SLD nomenclatures and liver fibrosis (Table 3). Patients with MASLD, MAFLD, MetALD, ALD, and MASLD‐Viral had unadjusted odds ratios (ORs) with 95% CIs of 2.69 (1.78, 4.07), 2.96 (1.99, 4.40), 3.09 (1.06, 8.94), 3.39 (1.16, 9.88), and 8.47 (1.75, 41.03), respectively. These findings suggest a significantly increasing trend in the odds of fibrosis development from MASLD to MASLD‐Viral, with the highest risk observed in the MASLD‐Viral group, which had over a ninefold increased odds compared with MASLD. After adjusting for potential confounders, including age, sex, CVD, history of cancer, and smoking status, the adjusted ORs (aORs) for MASLD, MAFLD, and MASLD‐Viral remained significantly associated with an increased odds of fibrosis, with aORs (95% CI) of 2.43 (1.56, 3.79), 2.63 (1.71, 4.06), and 7.59 (1.54, 37.47), respectively. These results confirmed that MAFLD is associated with a notably higher odds of liver fibrosis than those of MASLD, with aORs suggesting a more than twofold increase in risk. Moreover, MASLD‐Viral had the strongest association, with more than sevenfold increase in odds over that of MASLD. These findings are consistent with those of the UKB and NHANES, reinforcing the robustness of our results.

**TABLE 3:** Logistics regression analysis of SLD nomenclatures with liver fibrosis in FHCS.

	Total	Cases with fibrosis	Unadjusted OR (95% CI)	*p* value	Adjusted OR (95% CI)	*p* value
Without hepatic steatosis	1514	44	1.00 (Reference)		1.00 (Reference)	
MAFLD	770	61	2.96 (1.99, 4.40)	<0.001	2.63 (1.71, 4.06)	<0.001
SLD						
MASLD	693	51	2.69 (1.78, 4.07)	<0.001	2.43 (1.56, 3.79)	0.003
MetALD	48	4	3.09 (1.06, 8.94)	0.039	2.24 (0.74, 6.78)	0.154
ALD	44	4	3.39 (1.16, 9.88)	0.026	2.51 (0.83, 7.61)	0.105
MASLD‐Viral	10	2	8.47 (1.75, 41.03)	0.008	7.59 (1.54, 37.47)	0.013

Results were adjusted for age, sex, smoking, cardiovascular disease, and history of cancer.

Abbreviations: SLD, steatotic liver disease; FHCS, BeijngFH Health Cohort Study; OR, odds ratio; CI, confidence interval; SLD, cardiovascular disease; MAFLD, metabolic dysfunction‐associated fatty liver disease; MASLD, metabolic dysfunction‐associated steatotic liver disease; MetALD, MASLD with increased alcohol intake; ALD, alcohol‐related liver disease; MASLD‐Viral, MASLD with chronic viral hepatitis.

### 2.3 Stratified analysis of the relationship between MAFLD and mortality

We evaluated the survival differences according to sex, age, race, smoking status, CVD, and cancer history in patients with MAFLD (Table S3) or MASLD (Table 4). The results showed that MAFLD and MASLD were associated with a generally consistent risk of cancer‐related mortality and all‐cause mortality across subgroups, and both were associated with a higher risk of cancer‐related mortality and all‐cause mortality. In addition, a significant interaction was observed between sex, race, smoking status, and all‐cause mortality (*p* for interaction < 0.05), indicating that these variables may collectively impact the risk of all‐cause mortality in a nuanced manner instead of operating independently.

**TABLE 4:** Stratified analysis of the relationship between MASLD and mortality in the UKB and NHANES.

Subgroup	UKB				NHANES			
	Total	Case	aHR (95% CI)	*p* for interaction	Total	Case	aHR (95% CI)	*p* for interaction
Overall	404,615		1.32 (1.27, 1.36)		12,946		1.32 (1.14, 1.52)	
**Cancer‐related mortality**								
Sex				0.001				0.232
Male	165,203	7348	1.24 (1.19, 1.30)		6,071	597	1.22 (1.00, 1.48)	
Female	239,412	7957	1.41 (1.34, 1.48)		6875	515	1.45 (1.18, 1.79)	
Race				0.602				0.221
White	381,074	14,801	1.32 (1.27, 1.36)		8537	747	1.39 (1.18, 1.64)	
Other	23,541	504	1.28 (1.07, 1.54)		4409	365	1.10 (0.82, 1.46)	
Age				0.001				0.376
<65	344,750	10,539	1.37 (1.31, 1.42)		10,991	769	1.35 (1.13, 1.60)	
≥65	59,865	4766	1.23 (1.15, 1.30)		1955	343	1.09 (0.85, 1.39)	
Smoke				<0.001				0.061
Never	232,573	6581	1.40 (1.33, 1.48)		6323	347	1.63 (1.29, 2.07)	
Previous	132,024	6058	1.35 (1.29, 1.43)		3,002	343	1.15 (0.91, 1.45)	
Current	40,018	2666	1.04 (0.96, 1.13)		3621	422	1.18 (0.90, 1.55)	
CVD				0.106				0.807
No	378,778	13,508	1.33 (1.28, 1.38)		12,167	1015	1.32 (1.13, 1.53)	
Yes	25,837	1797	1.20 (1.09, 1.32)		779	97	1.14 (0.73, 1.79)	
Cancer				0.211				0.639
No	363,052	11,327	1.33 (1.28, 1.38)		12,258	958	1.28 (1.10, 1.49)	
Yes	41,563	3978	1.25 (1.17, 1.34)		688	154	1.34 (0.91, 1.97)	
**All‐cause mortality**								
Sex				<0.001				0.006
Male	165,203	16,700	1.26 (1.23, 1.30)		6071	2374	1.29 (1.17, 1.42)	
Female	239,412	14,389	1.57 (1.51, 1.62)		6875	2227	1.52 (1.38, 1.68)	
Race				0.275				0.803
White	381,074	29,858	1.39 (1.35, 1.42)		8537	3112	1.39 (1.28, 1.51)	
Other	23,541	1231	1.46 (1.30, 1.63)		4409	1489	1.38 (1.21, 1.58)	
Age				<0.001				<0.001
<65	344,750	20,302	1.47 (1.43, 1.51)		10,991	2807	1.58 (1.45, 1.73)	
≥65	59,865	10,787	1.27 (1.22, 1.32)		1955	1794	1.06 (0.95, 1.17)	
Smoke				<0.001				<0.001
Never	232,573	13,051	1.49 (1.44, 1.55)		6323	1714	1.59 (1.43, 1.77)	
Previous	132,024	12,368	1.45 (1.40, 1.51)		3002	1464	1.16 (1.03, 1.30)	
Current	40,018	5670	1.06 (1.00, 1.13)		3621	1423	1.48 (1.29, 1.69)	
CVD				0.006				0.375
No	378,778	25,656	1.40 (1.37, 1.44)		12,167	3954	1.41 (1.31, 1.52)	
Yes	25,837	5433	1.29 (1.22, 1.37)		779	647	1.15 (0.97, 1.36)	
Cancer history				<0.001				0.843
No	363,052	24,900	1.41 (1.37, 1.44)		12,258	4130	1.38 (1.29, 1.49)	
Yes	41,563	6189	1.30 (1.23, 1.37)		688	471	1.31 (1.05, 1.63)	

Results were obtained with Cox proportional hazards. Models were adjusted for age (category), sex, race, cigarette smoking, CVD, history of cancer, and Fib‐4.

Abbreviations: aHR, adjusted hazard ratio; CI, confidence interval; CVD, cardiovascular disease; Fib‐4, fibrosis‐4 index; MASLD, metabolic dysfunction‐associated steatotic liver disease; UKB, UK Biobank; NHANES, National Health and Nutrition Examination Survey.

### 2.4 Association of diabetes with outcomes in those with MAFLD or MAFLD

We evaluated the effect of diabetes on the risk of liver cirrhosis, liver cancer, and all‐cause and cancer‐related mortality in patients with MAFLD or MASLD (Table 5). In individuals with MAFLD and MASLD, diabetes significantly increases the risk of cirrhosis, liver cancer, and all‐cause mortality. Data from the UKB reveal starkly elevated aHRs for patients with diabetes—3.33 (2.99, 3.71) for cirrhosis, 3.40 (2.85, 4.06) for liver cancer, and 1.94 (1.87, 2.00) for MAFLD‐related mortality. For MASLD, the corresponding aHRs are 3.45 (3.00, 3.95), 3.29 (2.64, 4.10), and 1.97 (1.89, 2.05). Similar trends are observed in the NHANES, where aHRs for all‐cause mortality are higher at 1.74 (1.55, 1.95) and 1.79 (1.58, 2.03) in diabetic patients with MAFLD and MASLD, respectively, than in nondiabetic individuals (Table 5). These results suggest that diabetes is a significant risk factor for adverse liver events and mortality in patients with fatty liver disease. Subsequently, we aimed to explore the role of antiglycemic medications (insulin, metformin, sulfonylureas, etc.) in this population.

**TABLE 5:** Association of diabetes with liver cirrhosis, liver cancer, cancer‐related mortality, and all‐cause mortality in MAFLD and MASLD.

Group 1 (MAFLD)	Total	Case	Rate^a^	aHR (95% CI)^b^	*p* value	Group 2 (MASLD)	Total	Case	Rate^a^	aHR (95% CI)^c^	*p* value
UKB (*N* = 177,106)						UKB (*N* = 117,744)					
**Cirrhosis**											
Nondiabetic MAFLD	156,540	1225	0.61	1.00 (Reference)		Nondiabetic MASLD	102,138	622	0.47	1.00 (Reference)	
Diabetic MAFLD	20,566	509	2.02	3.33 (2.99, 3.71)	<0.001	Diabetic MASLD	15,606	331	1.73	3.45 (3.00, 3.95)	<0.001
**Liver cancer**											
Nondiabetic MAFLD	156,540	417	0.21	1.00 (Reference)		Nondiabetic MASLD	102,138	239	0.18	1.00 (Reference)	
Diabetic MAFLD	20,566	195	0.77	3.40 (2.85, 4.06)	<0.001	Diabetic MASLD	15,606	129	0.67	3.29 (2.64, 4.10)	<0.001
**Cancer‐related mortality**											
Nondiabetic MAFLD	156,540	7246	3.59	1.00 (Reference)		Nondiabetic MASLD	102,138	4602	3.49	1.00 (Reference)	
Diabetic MAFLD	20,566	1482	5.86	1.45 (1.37, 1.54)	<0.001	Diabetic MASLD	15,606	1,110	5.78	1.47 (1.38, 1.57)	<0.001
**All‐cause mortality**											
Nondiabetic MAFLD	156,540	14,754	7.30	1.00 (Reference)		Nondiabetic MASLD	102,138	9349	7.09	1.00 (Reference)	
Diabetic MAFLD	20,566	4286	16.95	1.94 (1.87, 2.00)	<0.001	Diabetic MASLD	15,606	3224	16.78	1.97 (1.89, 2.05)	<0.001
NHANES (*N* = 2773)						NHANES (*N* = 2452)					
**Cancer related mortality**											
Nondiabetic MAFLD	2033	215	4.57	1.00 (Reference)		Nondiabetic MASLD	1802	190	4.50	1.00 (Reference)	
Diabetic MAFLD	740	76	5.39	1.12 (0.86, 1.46)	0.411	Diabetic MASLD	650	65	5.22	1.11 (0.83, 1.48)	0.485
**All‐cause mortality**											
Nondiabetic MAFLD	2033	863	18.33	1.00 (Reference)		Nondiabetic MASLD	1802	729	17.25	1.00 (Reference)	
Diabetic MAFLD	740	481	34.13	1.74 (1.55, 1.95)	<0.001	Diabetic MASLD	650	420	33.72	1.79 (1.58, 2.03)	<0.001

Results were obtained with Cox proportional hazards model and given as aHR with 95% CI.

Abbreviations: aHR,  adjusted hazard ratio; CI,  confidence interval; CVD,  cardiovascular disease; Fib‐4 , fibrosis‐4 index; MAFLD,  metabolic dysfunction‐associated fatty liver disease; MASLD,  metabolic dysfunction‐associated steatotic liver disease.

^a^ Incidence rate per 1000 person years.

^b^ Models were adjusted for age (category), sex, race, cigarette smoking, alcohol intake, Fib‐4, CVD, history of cancer, and viral hepatitis.

^c^ Models were adjusted for age (category), sex, race, cigarette smoking, CVD, history of cancer, and Fib‐4.

### 2.5 Association of antiglycemic medications with outcomes in those with MAFLD or MAFLD

The effects of antiglycemic medications on the prognosis of diabetes‐associated MAFLD and MASLD were evaluated in the UKB cohort (Table 6). Commonly used medications included metformin monotherapy (MAFLD: 26.3%, MASLD: 26.7%), combination of metformin and sulfonylureas (MAFLD: 12.3%, MASLD: 12.9%), insulin with metformin (MAFLD: 7.7%, MASLD: 8.6%), insulin alone (MAFLD: 6.0%, MASLD: 6.1%), sulfonylureas alone (MAFLD: 2.8%, MASLD: 2.7%), and the triple combination of insulin, metformin, and sulfonylureas (MAFLD: 1.4%, MASLD: 1.6%). After adjusting for age; sex; race; body mass index (BMI); smoking and alcohol consumption statuses; Fib‐4 score; glycated hemoglobin level; a history of hypertension, CVD, cancer history, viral hepatitis; and aspirin or statin use, we observed that insulin, sulfonylureas, and their combination with metformin were associated with a higher risk of cirrhosis and all‐cause mortality in patients with MAFLD or MASLD. Metformin was associated with a lower risk of all‐cause and cancer‐related mortality; however, this association did not reach statistical significance. Moreover, the combinations of insulin with metformin and metformin with sulfonylureas were associated with an increased risk of liver cancer in patients with MAFLD and MASLD (Table 6). These results indicate that the use of antiglycemic agents in patients with diabetes and fatty liver disease may not reduce the risk of adverse liver events or mortality. Instead, the prevention of diabetes may be crucial for improving patient outcomes in this population. However, these findings require validation through rigorous studies in external cohorts, and further research is required to elucidate the underlying mechanisms.

**TABLE 6:** Association of antiglycemic medication use with risk of cirrhosis, liver cancer, cancer‐related death, and all‐cause mortality in MAFLD and MASLD patients in the UK Biobank.

	MAFLD (*N* = 18,617)			MASLD (*N* = 14,017)		
	Case/total	aHR (95% CI)^a^	*p* value	Case/total	aHR (95% CI)^b^	*p* value
**Cirrhosis**						
No drug	158/8085	1.00 (Reference)		88/5776	1.00 (Reference)	
Insulin	33/1118	1.82 (1.22, 2.71)	0.003	21/862	1.72 (1.03, 2.88)	0.037
Metformin	115/4898	1.28 (0.99, 1.66)	0.058	72/3751	1.35 (0.97, 1.89)	0.078
Sulfonylureas	21/521	2.32 (1.45, 3.72)	<0.001	15/384	2.92 (1.67, 5.11)	<0.001
Insulin and metformin	52/1442	2.24 (1.59, 3.15)	<0.001	41/1,207	2.47 (1.64, 3.71)	<0.001
Insulin, metformin, and sulfonylureas	11/261	2.90 (1.55, 5.41)	<0.001	9/225	3.13 (1.55, 6.33)	0.001
Metformin and sulfonylureas	62/2292	1.58 (1.15, 2.18)	0.004	46/1812	1.87 (1.27, 2.75)	0.001
**Liver cancer**						
No drug	63/8085	1.00 (Reference)		36/5776	1.00 (Reference)	
Insulin	9/1118	1.37 (0.67, 2.80)	0.390	6/862	1.34 (0.55, 3.26)	0.514
Metformin	33/4898	1.12 (0.72, 1.74)	0.616	24/3,751	1.31 (0.76, 2.24)	0.330
Sulfonylureas	5/521	1.44 (0.58, 3.61)	0.435	3/384	1.38 (0.42, 4.53)	0.597
Insulin and metformin	19/1442	2.29 (1.31, 4.01)	0.004	15/1207	2.39 (1.23, 4.67)	0.010
Insulin, metformin, and sulfonylureas	2/261	1.40 (0.34, 5.80)	0.645	1/225	0.88 (0.12, 6.51)	0.900
Metformin and sulfonylureas	46/2292	3.17 (2.08, 4.82)	<0.001	33/1812	3.28 (1.94, 5.55)	<0.001
**Cancer‐related mortality**						
No drug	567/8085	1.00 (Reference)		405/5,776	1.00 (Reference)	
Insulin	84/1118	1.09 (0.86, 1.40)	0.477	66/862	1.10 (0.84, 1.46)	0.488
Metformin	311/4898	0.93 (0.80, 1.08)	0.331	235/3751	0.91 (0.77, 1.08)	0.299
Sulfonylureas	37/521	0.91 (0.64, 1.29)	0.592	22/384	0.71 (0.45, 1.12)	0.138
Insulin and metformin	115/1442	1.14 (0.92, 1.43)	0.226	97/1,207	1.10 (0.86, 1.41)	0.435
Insulin, metformin, and sulfonylureas	20/261	1.12 (0.70, 1.78)	0.633	16/225	0.97 (0.58, 1.64)	0.921
Metformin and sulfonylureas	192/2292	1.24 (1.04, 1.47)	0.017	141/1812	1.12 (0.91, 1.37)	0.295
**All‐cause mortality**						
No drug	1409/8085	1.00 (Reference)		992/5776	1.00 (Reference)	
Insulin	384/1118	1.92 (1.71, 2.17)	<0.001	304/862	1.95 (1.70, 2.24)	<0.001
Metformin	824/4898	0.95 (0.87, 1.04)	0.282	610/3751	0.94 (0.85, 1.05)	0.271
Sulfonylureas	151/521	1.50 (1.26, 1.78)	<0.001	100/384	1.30 (1.05, 1.61)	0.016
Insulin and metformin	437/1442	1.58 (1.41, 1.78)	<0.001	366/1,207	1.53 (1.35, 1.75)	<0.001
Insulin, metformin, and sulfonylureas	86/261	1.70 (1.35, 2.13)	<0.001	74/225	1.61 (1.26, 2.07)	<0.001
Metformin and sulfonylureas	552/2292	1.31 (1.18, 1.46)	<0.001	410/1812	1.23 (1.09, 1.39)	<0.001

Results were obtained with Cox proportional hazards model and given as aHR with 95% CI.

Abbreviations: aHR, adjusted hazard ratio; CI, confidence interval; CVD, cardiovascular disease; Fib‐4, fibrosis‐4 index; MAFLD, metabolic dysfunction‐associated fatty liver disease; MASLD, metabolic dysfunction‐associated steatotic liver disease; ALD, alcohol‐related liver disease.

^a^ Models were adjusted for age (category), sex, race, BMI, cigarette smoking, alcohol intake, Fib‐4, glycated hemoglobin, hypertension, CVD, history of cancer, viral hepatitis, exposure to aspirin and exposure to statin.

^b^ Model were adjusted for age (category), sex, race, BMI, cigarette smoking, Fib‐4, glycated hemoglobin, hypertension, CVD, history of cancer, exposure to aspirin and exposure to statin.

## 3 DISCUSSION

In this study, we examined the prognostic and associative effects of the former term, MAFLD, and the updated terms, namely MASLD, MetALD, ALD, and MASLD‐Viral in two prospective cohorts of the UKB and NHANES and a case–control study, FHCS. After adjusting for age, sex, race, smoking, Fib‐4, CVD, and cancer history, MAFLD, MASLD, MetALD, ALD, and MASLD‐Viral were associated with an increased risk of liver cirrhosis, liver cancer, and all‐cause and cancer‐related mortalities compared with non‐SLD. Additionally, the MAFLD group was adjusted for alcohol consumption and viral hepatitis. Compared with MASLD, MAFLD identified a greater number of individuals with higher risks of liver cirrhosis and liver cancer in the UKB cohort and higher risks of all‐cause and cancer‐related mortalities in the NHANES cohort. In the case–control study from FHCS, we found that MAFLD was associated with higher odds of fibrosis than MASLD. Diabetes increases the risk of liver cirrhosis, liver cancer, all‐cause mortality, and cancer‐related mortality in MAFLD or MASLD; furthermore, three conventional antiglycemic medications may increase the risk of cirrhosis and all‐cause mortality in diabetes‐associated MAFLD and MASLD. Although metformin reduced the risk of all‐cause and cancer‐related mortality in these patients, the differences were not statistically significant. Nevertheless, the combination of metformin with insulin or sulfonylureas has been linked to an increased risk of liver cancer in these individuals. These findings emphasize that the new SLD nomenclatures for disease subdivision will aid in making clinical decisions regarding therapeutic and prophylactic options, especially highlighting the importance of preventing diabetes in patients with MAFLD or MASLD because antidiabetic medications increase the risks of adverse liver outcomes and reduces survival in patients with diabetes‐associated MAFLD and MASLD.

The redefinition of SLD has resulted in significant changes in patient classification, which may lead to changes in disease prognosis and therapeutic measures. In the NHANES cohort, 19.3, 20.7, and 18.3% of participants met the diagnostic criteria for NAFLD, MAFLD, and MASLD, respectively, with an overall co‐prevalence of 17.7%. Notably, 91.8% (2390/2604) of patients with NAFLD met the diagnostic criteria for MAFLD, whereas 95.3% (2481/2604) of those with NAFLD met the criteria for MASLD. Compared with MAFLD, MASLD reclassifies fewer individuals with NAFLD, indicating that the differences between MASLD and NAFLD are smaller than those between MAFLD and NAFLD. Previous research on NAFLD may apply to MASLD.^16^ Only 85.1% (2389/2806) of patients with MAFLD met the diagnostic criteria for MASLD. Additionally, 6.6% (185/2806) of those with MAFLD met the criteria for MetALD, and 6.2% (175/2806) met the criteria for ALD. The shift from MAFLD to MASLD is largely due to a considerable portion of the population being reclassified from MAFLD to new categories, such as MetALD, ALD, and MASLD‐Viral. In the UKB cohort, 38.1% (177,106/464,556) and 25.3% (117,744/464,556) of participants met the diagnostic criteria for MAFLD and MASLD, respectively. Only 66.3% (117,469/177,106) of patients with MAFLD met the diagnostic criteria for MASLD, whereas 22.5% (39,863/177,106) met the diagnostic criteria for MetALD and 11.0% (19,561/177,106) met the diagnostic criteria of ALD. The higher prevalence of SLD in the UKB cohort than that in the NHANES cohort may be explained by several factors. (1) Participants in the UKB cohort were older than those in the NHANES cohort (mean, 56.5 vs. 43.9 years). As shown in Figure 2A–D, we found that the prevalence of MAFLD and MASLD increased with age. Previous studies have also shown that 50–59 and ≥60 years of age are independent risk factors for NAFLD.^17^ (2) The diagnostic criteria, as outlined in the methods section, are slightly different. (3) The participants were recruited in the NHANES III cohort during 1988–1994, whereas those in the UKB cohort were recruited during 2006–2010. The prevalence of MASLD and liver complications related to NAFLD has increased over the past decade.^18^

Although MAFLD and MASLD are associated with a poor prognosis, studies have also reported conflicting findings.^11^, ^19^ Some researchers argue that MAFLD has no impact on all‐cause mortality, whereas others have suggested that MASLD does not affect cancer‐related mortality.^14^ In this study, both MAFLD and MASLD exhibited increased risks of all‐cause and cancer‐related mortalities independently. Notably, MetALD was associated with the lowest risk of all‐cause and cancer‐related mortalities among the four subtypes of SLD in the UKB and NHANES cohorts; however, MetALD was not significantly associated with cancer‐related mortality in the NHANES cohort. In addition, the risks of all‐cause and cancer‐related mortalities were higher in MAFLD than in MASLD in the NHANES cohort but not in the UKB cohort. This inconsistency is caused by the differences in the covariates adjusted and the discrepancy in defining MetALD between the two cohorts. This discrepancy in the definition of MetALD might be responsible for the different risks of all‐cause and cancer‐related mortalities between the UKB and NHANES cohorts. In the UKB cohort, MetALD is characterized by 30–60 and 20–50 g/day of alcohol consumption for men and for women, respectively, whereas in the NHANES cohort, it is characterized by 2–4 standard drinks per day for men and 1–3 standard drinks per day for women. In a prospective cohort study, severe alcohol intake was defined as consumption of >24 and >36 g/day alcohol for women and men, respectively.^20^ Due to the heterogeneity of the databases, complete standardization of alcohol intake is difficult, and future studies are necessary to further validate our findings with similar alcohol intake. Notably, MASLD‐Viral was associated with the highest risk of liver cirrhosis, liver cancer, all‐cause mortality, and cancer‐related mortality in both cohorts. Some studies have suggested that the presence of hepatic steatosis may suppress HBV viral activity, potentially leading to attenuated liver injury.^21^ In contrast, coincidence of fatty liver and metabolic dysfunction may increase the risk of developing adverse liver outcomes in HBV‐infected patients.^22^, ^23^ In this study, we confirmed that concurrent viral hepatitis with MASLD greatly increased the risk of liver cirrhosis, liver cancer, all‐cause mortality, and cancer‐related mortality, highlighting the need to control viral hepatitis in this population. Additionally, diabetes exacerbated adverse outcomes in individuals with MAFLD or MASLD across the two distinct cohorts. Consistent with the findings of earlier research, the risks of hepatocellular carcinoma (HCC) and mortality were notably elevated in diabetic patients with MAFLD compared with their nondiabetic counterparts.^24^, ^25^, ^26^ These earlier findings were primarily based on studies including Asian populations. Our study extends these conclusions to large European and U.S. cohorts, further confirming the association between diabetes and poor prognosis in patients with MAFLD.

Prior research suggests that antiglycemic medications, such as metformin and thiazolidinediones, which enhance insulin sensitivity, can lower the chances of developing HCC. However, the use of insulin or sulfonylureas drugs, which elevate circulating insulin levels, has been associated with an increased risk of HCC.^27^, ^28^, ^29^, ^30^ In our study, we found that metformin monotherapy was associated with an increased risk of cirrhosis but was not associated with liver cancer, overall mortality, or cancer‐related mortality. However, metformin plus insulin or metformin plus sulfonylureas increased the risk of liver cancer in patients with diabetes‐associated MAFLD or MASLD. Sulfonylureas, known as insulin secretagogues, may promote carcinogenesis by increasing the activity of insulin‐like growth factor‐1 (IGF‐1), which stimulates cell proliferation.^31^ Insulin and IGF‐1 receptors, which are abundantly present on cancer cells, facilitate the activation of key signaling pathways such as mitogen‐activated protein kinase and mammalian target of rapamycin.^32^ The activation of these pathways is pivotal in the development and progression of cancer. Our research underscores the significance of diabetes prevention in individuals with fatty liver disease, owing to its widespread occurrence, unfavorable outcomes, and uncertain results of antiglycemic drugs.^33^, ^34^ Although metformin is commonly prescribed as the primary treatment for type 2 diabetes and has been proposed to enhance survival rates in diabetic patients with cirrhosis,^35^ evidence supporting its efficacy remains inconclusive. Randomized controlled trials have shown no significant efficacy of metformin in reducing hepatic steatosis.^36^, ^37^ Moreover, both European and United States guidelines for diagnosing and treating NAFLD have concluded that there is insufficient evidence supporting the use of metformin for this condition.^38^, ^39^ For patients with MASLD, preventing diabetes is more important than treating it. Guidelines recommend starting with weight loss for overweight or obese patients with type 2 diabetes and fatty liver.^39^, ^40^ To achieve improvements in blood sugar control and steatosis, it is advisable to reduce body weight by at least 5%, with further weight loss (>7%) associated with greater enhancements in serum glycemic control and metabolic indicators, as well as a notable reduction in inflammation and fibrosis in fatty liver.^38^, ^41^ The guidelines for both type 2 diabetes and NAFLD suggest engaging in approximately 150–200 min of physical activity per week.^41^ Therefore, public health prevention programs should be implemented from the early stages of SLD to prevent the onset of diabetes.^42^

Our study has several limitations. First, our study utilized the fatty liver index (FLI) instead of the gold standard methods such as ultrasound examination or liver biopsy for diagnosing hepatic steatosis to define SLD in the UKB cohort. However, the accuracy of FLI has been validated, and it is recommended for diagnosing MAFLD.^4^ Second, alcohol consumption and metabolic metrics were only measured at baseline, and we were unable to track changes in these metrics during the follow‐up period, during which status may have changed. Third, owing to sample size limitations, we were unable to perform separate analyses for MASLD–HBV and MASLD–HCV to further compare the specific differences between the two viral hepatitis types. Finally, the limitations of the UKB prevented us from obtaining information on the dose and duration of antiglycemic medications, which is a critical aspect in assessing their impact on patient outcomes. Future research should address these limitations by using a combination of diagnostic methods, including the FLI along with ultrasound or liver biopsy,^43^ implementing stricter criteria for the classification of MAFLD, including cohorts that provide detailed information on medication dosage and duration, and monitoring changes in metabolic status and alcohol consumption throughout the study.

In conclusion, MAFLD identified a greater number of individuals with a higher risk of liver‐related adverse outcomes and mortality than MASLD, whereas MASLD and other SLD subtypes effectively delineated a cohort with intervention‐worthy risk factors. Diabetes is a significant contributor to cancer and mortality risks in patients with MASLD. The use of antiglycemic medications in patients with diabetes‐associated MAFLD or MASLD warrants careful consideration.

## 4 MATERIALS AND METHODS

### 4.1 Study population

The study included two cohorts from the UKB and NHANES and a case–control study from the FHCS. The UKB is a large‐scale, prospective cohort study conducted from 2006 to 2010, including over 500,000 participants aged 40–69 years, recruited across 22 centers in Scotland, England, and Wales.^44^ This extensive project combined baseline data, genotypic information, and national medical records for a complete long‐term study. This study is officially registered with the UK Biobanking Resource Center under application number 101971. The UKB obtained approval from the North West Multicenter Research Ethics Committee, the National Health and Social Care Information Management Board for England and Wales, and the Scottish Community Health Index Advisory Group. The NHANES, managed by the US National Center for Health Statistics (NCHS), is a population‐based survey that aims to track the health and nutrition of the US civilian population using a complex, multistage approach.^45^ NHANES III, conducted between 1988 and 1994, is the sole survey phase that includes liver ultrasound information for adults aged between 20 and 74 years. It is imperative to highlight that all participants provided informed consent. The NHANES was approved by the Research Ethics Review Board of the NCHS. After excluding participants with missing data for key variables (NAFLD, MAFLD, SLD, and mortality), we conducted multiple imputations using the random forest algorithm within the multiple imputation by chained equations framework to deal with missing data for covariates, such as race, smoking status, BMI, Fib‐4 score, alcohol consumption status, hypertension, CVD, and cancer history in the two cohorts. The FHCS is a community‐based cohort study that began in 2022 with the aim of establishing a risk stratification assessment system for MAFLD. The study was conducted including participants who underwent health checkups at the outpatient clinic of the Beijing Friendship Hospital branch center. Baseline information, including demographic data, clinical history, physical examination, laboratory results, and imaging tests, was collected and followed up annually. The FHCS is registered at ClinicalTrials.gov (NCT05546086. The Capital Medical University approved the study (2022‐P2‐025‐01), and all participants provided informed consent.

### 4.2 Definition of baseline SLD nomenclatures

In the UKB cohort, SLD was defined by an FLI score of ≥60, due to the absence of liver histology and imaging data.^46^ The FLI, a composite scoring system that assesses waist circumference (WC), γ‐glutamyl transferase level, triglycerides level, and body mass index, was utilized identify steatohepatitis. In contrast, the presence of moderate or severe hepatic steatosis was used as a diagnostic criterion for SLD in the NHANES cohort. In the FHCS cohort, SLD was defined as hepatic steatosis diagnosed using ultrasonography, regardless of severity. Liver fibrosis is defined as a liver stiffness measurement greater than 7.4 kPa on FibroScan. MAFLD is diagnosed when SLD is accompanied by either overweight or obesity (BMI ≥ 25 kg/m^2^), type 2 diabetes, or at least two metabolic abnormalities such as increased WC, arterial hypertension, hypertriglyceridemia, low HDL cholesterol, prediabetes, subclinical inflammation, or insulin resistance.^5^ Due to the absence of serum insulin data in the UKB, the assessment of insulin resistance was not conducted. MASLD was defined as the presence of SLD and one or more of the following cardiometabolic risk factors: (1) BMI ≥ 25 kg/m^2^ or WC ≥ 90 cm for men or ≥80 cm for women; (2) HbA1c ≥ 5.7% (39 mmol/mol), fasting serum glucose ≥ 5.6 mmol/L (≥100 mg/dL), 2‐h post‐load glucose levels ≥7.8 mmol/L (≥140 mg/dL), a diagnosis of type 2 diabetes, or use of antiglycemic drugs; (3) blood pressure ≥ 130/85 mmHg or use of antihypertensive drugs; (4) triglycerides ≥ 1.70 mmol/L (150 mg/dL) or use of lipid‐lowering drugs; (5) HDL‐cholesterol ≤ 1.0 mmol/L (40 mg/dL) for men and ≤1.3 mmol/L (50 mg/dL) for women or use of lipid‐lowering drugs.^6^ MASLD does not include increased or excessive alcohol intake or chronic viral hepatitis (hepatitis B or hepatitis C). Detailed diagnostic criteria for MAFLD and MASLD are presented in Table S4. Among individuals with SLD and one or more cardiometabolic risk factors, self‐reported alcohol intake was categorized as a criterion for MetALD if it ranged from 20 to 50 g/day for women and 30 to 60 g/day for men. Patients with SLD is considered having ALD if they have at least one cardiometabolic risk factor and self‐report consuming more than 50 g/day of alcohol for women and more than 60 g/day of alcohol for men or it they do not have cardiometabolic risk factors and self‐report consuming more than 20 g/day of alcohol for women and more than 30 g/day of alcohol for men.^19^ MASLD‐Viral hepatitis was diagnosed if the patient had metabolic dysfunction and viral hepatitis.

### 4.3 Assessment of antiglycemic medication use

Medication data were sourced from the UKB under Field ID 20003. This category included information on prescription medications obtained through interviews with trained nurses who recorded the type and dosage of each medication. Participants who reported regular use of prescription medications were identified via a pop‐up notification on the interviewer's computer screen. The medications typically included metformin, sulfonylureas, glitazones, and insulin. This category covered data on any medication administered on a regular basis, either weekly, monthly, or other intervals. Glucose‐lowering medications were classified into several groups: insulin, metformin, sulfonylurea, insulin/metformin combinations, insulin/metformin/sulfonylurea combinations, and metformin/sulfonylurea combinations. A detailed breakdown of these medication categories is presented in Table S5.

### 4.4 Assessment of covariates

Blood pressure was measured by trained staff following standard procedures; HbA1c was measured using high‐performance liquid chromatography.^47^ The prevalence of hypertension at baseline was determined based on self‐reported hypertension or the use of antihypertensive medication, systolic blood pressure ≥130 mmHg or diastolic blood pressure ≥85 mmHg. WC was measured by a trained staff member using a steel tape measure directly above the iliac crest according to a standardized technique. BMI was calculated by dividing the weight in kilograms by the square of height in square meters. Demographic information, including age, sex, race, education, smoking status, alcohol consumption status, and medical history (chronic viral hepatitis, hypertension, CVD, and cancer history), was obtained through baseline questionnaires or verbal interviews. Additionally, in the UKB, the occurrences of CVD and cancer were identified using ICD‐10‐coded or ICD‐9‐coded hospital admission records, cancer registries, and self‐reported data (Table S6). The Fib‐4 index was calculated based on platelet count, age, aspartate aminotransferase level, and alanine aminotransferase level.^48^ In the UKB, participants were queried about their current smoking status, with “nonsmoking” indicating a healthy status. In the NHANES cohort, participants were queried about their lifetime smoking history (≥100 cigarettes) and current smoking status.

In the UKB, pure alcohol intake (gram) was calculated by multiplying the average number of alcoholic beverages consumed per week (or month) by the average number of grams of alcohol in each beverage, following the UK Food Standards Agency guidelines (https://www.drinkaware.co.uk/low‐risk‐drinking‐guidelines) (Table S7). The total amount of alcohol consumption was then divided by 7 (or 30) to calculate the average daily alcohol intake (g/day). Participants who reported that they had never previously or currently consumed alcohol as lifetime nondrinkers. Men who consumed <30, 30–60, and >60 g of pure alcohol per day were defined as mild, moderate, and severe drinkers, respectively. Women who consumed < 20, 20–50, and >50 g of pure alcohol per day were defined as mild, moderate, and severe drinkers, respectively. In the NHANES, alcohol consumption over the past year was assessed using a food frequency questionnaire, and alcohol intake was calculated based on the reported frequency and portion sizes of beer, wine, and liquor consumption. Mild alcohol consumption was defined as consumption not exceeding two standard drinks per day for men and one standard drink per day for women (one standard drink = 14 g of ethanol) (Table S7). Moderatealcohol consumption was defined as the consumption two to four standard drinks per day for men and one to three standard drink per day for women. Severe alcohol consumption was defined as consumption exceeding four standard drinks per day for men and three standard drinks per day for women.

### 4.5 Assessment of outcomes

In the UKB, follow‐up data on liver‐related events and mortality were obtained through electronic links to hospital admission and cancer registries in England, Wales, and Scotland. The study outcomes included cirrhosis, liver cancer, and all‐cause mortality, categorized using the International Classification of Diseases (ICD‐10 and ICD‐9), as detailed in Table S5. Liver cancer events were identified using ICD‐10 code C22 or ICD‐9 codes 155.0‐155.3. In the UKB, dates of death were obtained from death certificates in files at the National Health Service Information Center (England and Wales) and the National Health Service Central Registry (Scotland). The time to event was computed as the duration from the participant's baseline enrollment date to the date of their first diagnosis of cirrhosis or liver cancer, or until the date of their last follow‐up visit (May 31, 2022), whichever came first. In the NHANES, no incidence of liver‐related events or site‐specific cancer‐related deaths were reported. The NCHS provides public‐use mortality data linked to the National Death Index with a cutoff date of December 31, 2019, mortality follow‐up data.^49^ NHANES uses and ICD‐10 to code the causes of death. Follow‐up duration was defined as the number of years between the date of the interview and the date of cancer death or all‐cause death. The follow‐up end date was December 31, 2019, for participants without events.

### 4.6 Statistical analysis

After excluding data wherein primary variables (dependent and outcome variables) were missing, we performed multiple imputations for the maximum retention of participants. For continuous variables, median and interquartile range were used for non‐normally distributed data, whereas mean and standard deviation were used for normally distributed data. Differences between groups were assessed using the *t*‐test and Wilcoxon rank‐sum test (Mann–Whitney *U* test). Categorical variables are presented as frequencies (percentages), and between‐group differences were evaluated using the chi‐square test. Hazard ratios and their corresponding 95% CIs were estimated using Cox proportional hazards modeling, whereas ORs were derived from logistic regression analysis to assess the association between SLD nomenclature and liver fibrosis. This study further explored the relationship between MAFLD and MASLD and the risk of all‐cause mortality and cancer‐related mortality across various subgroups categorized by factors such as sex, age, race, smoking status, CVD, and cancer history. Statistical analyses were performed using the STATA 14.0 (Stata Corporation, College Station, TX, USA) and R software (version 4.1.0). Two‐sided *p* values of 0.05 were considered statistically significant for all tests.

## AUTHOR CONTRIBUTIONS

H. X. Z., L. T. F., Z. Y. Y., H. Y., and G. W. C. conceived the study and designed the research. H. X. Z., L. T. F., Z. Y. Y., and X. Y. Z. contributed to the acquisition, analysis, or interpretation of data. L. T. F., H. X. Z., and Z. Y. Y. drafted the manuscript. G. W. C. and H. Y. critically revised the manuscript for important intellectual content. H. X. Z., Z. Y. Y., L. T. F., and X. Y. Z. performed the statistical analysis. H. S. C., P. Y. X., Z. Y. N., Z. L., Z. S. L., J. Y. Z., W. B. L., and C. X. J. provided administrative, technical, or material support. G. C. and H. Y. supervised the study. All authors have read and approved the final manuscript.

## CONFLICT OF INTEREST STATEMENT

All authors declare no conflicts of interest.

## ETHICS STATEMENT

All participants provided written consent to participate in the UKB and NHANES III studies. As this study only extracted data from the UKB and NHANES website for secondary data analysis, further ethical approval is not required. Beijing Capital Medical University approved the ethical situation of BeijngFH Health Cohort Study (2022‐P2‐025‐01), and all participants provided informed consent.

## Supporting information



Supporting information

## Data Availability

This study was conducted utilizing resources from the UK Biobank (Application Number 101971), the NHANES III cohort study, available at www.cdc.gov/nchs/nhanes, and the BeijngFH Health Cohort Study (NCT05546086).
