# Supplementary material for: Prognostic and predictive effects of new steatotic liver disease nomenclatures: a large population‐based study
Source: MedComm (2020). 2025 Feb 13;6(2):e70087. doi: 10.1002/mco2.70087 (PMC11822458; doi:10.1002/mco2.70087)
Supplement: Supplementary file 1 — Supporting information [file MCO2-6-e70087-s001.docx]

**Supplementary Materials**

**Prognostic and Predictive Effects of New Steatotic Liver Disease** **Nomenclatures: a large population-based study**

Huixian Zeng^1,2,3,4,5#^, Letian Fang^2,3,4#^, Zhiyu Yang,^1,2,3,4,6#^, Xinyu Zhao^7#^, Hongsen Chen^2,3,4^, Puyi Xing^1,2,3,4^, Zheyun Niu^8^, Zheng Li^2,3,4^, Zishuai Li^2,3,4^, Jiayi Zhao^2,3,4^, Wenbin Liu^2,3,4^, Chunxia Jing^1^, Hong You^9*^, Guangwen Cao^2,3,4*^

^1^ Department of Epidemiology, School of Medicine, Jinan University, Guangzhou, 510632, Guangdong, China

^2^ Key Laboratory of Biological Defense, Ministry of Education, Second Military Medical University, Shanghai, 200433, China

^3^ Shanghai Key Laboratory of Medical Bioprotection, Second Military Medical University, Shanghai, 200433, China

^4^ Department of Epidemiology, Second Military Medical University, Shanghai, 200433, China

^5^ Jiading District Center for Disease Control and Prevention, Shanghai, 201800, China

^6^ Shanghai Municipal Center for Disease Control and Prevention, Shanghai, 200336, China

^7^ Clinical Epidemiology & EBM Unit, Beijing Friendship Hospital, Capital Medical University; National Clinical Research Center for Digestive Diseases, 100050, Beijing, China

^8^ Shanghai East Hospital, Key Laboratory of Arrhythmias, Ministry of Education, Tongji University School of Medicine, Tongji University, Shanghai 200120, China

^9^ Liver Research Center, Beijing Friendship Hospital, Capital Medical University; State Key Lab of Digestive Health, National Clinical Research Center of Digestive Diseases, Beijing, 100050, China

^#^Joint first authors

**^*^Correspondence:**

Guangwen Cao, M.D., Ph.D.

Department of Epidemiology, Second Military Medical University, No. 800 Xiangyin Rd., Shanghai 200433, China.

Telephone & Fax: +86-21-81871060. E-mail address: [gcao@smmu.edu.cn](mailto:gcao@smmu.edu.cn)

Hong You, M.D., Ph.D.

Liver Research Center, Beijing Friendship Hospital, Capital Medical University, No. 95 Yong’an Rd., Beijing 100050, China

Telephone: +86‐10‐631390. E-mail address: [youhongliver@ccmu.edu.cn](mailto:youhongliver@ccmu.edu.cn)

**Contents**

[Figure S1. The proportion of participants under SLD nomenclatures 3](#_Toc180483322)

[Figure S2. The comparison of metabolic dysfunction prevalence in participants with and without SLD 4](#_Toc180483323)

[Figure S3. Kaplan-Meier analysis of cancer-related and all-cause mortalities in participants with NAFLD, MAFLD, and MASLD 5](#_Toc180483324)

[Table S1. Detailed baseline characteristics of study participants with MAFLD 7](#_Toc180483325)

[Table S2. Detailed baseline characteristics of study participants with MASLD 9](#_Toc180483326)

[Table S3. Relationship between MAFLD and mortality: stratified analysis. 12](#_Toc180483327)

[Table S4. Definition factors of MAFLD and MASLD 14](#_Toc180483328)

[Table S5. Codes used to identify medications in the UKB cohort 17](#_Toc180483329)

[Table S6. ICD-10 and ICD-9 codes used to determine liver disease and medical history 22](#_Toc180483330)

[Table S7. Calculation of pure alcohol intake in the UKB cohort 23](#_Toc180483331)


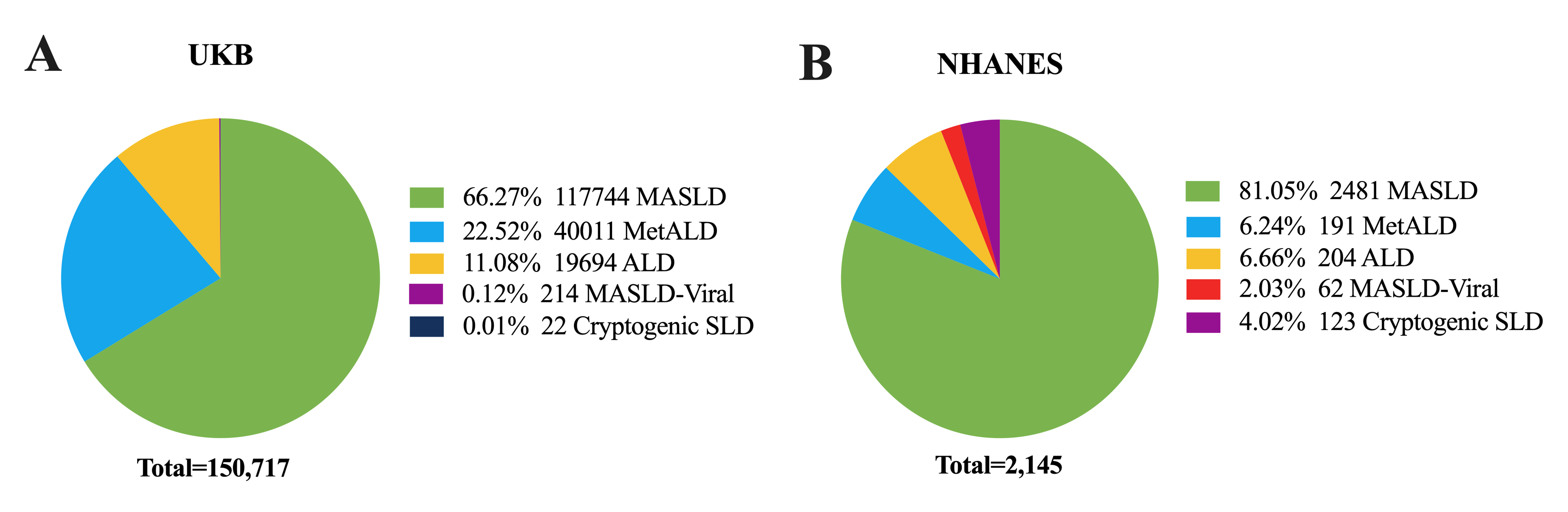


### Figure S1. The proportion of participants under SLD nomenclatures

(A) UKB. (B) NHANES. SLD=steatotic liver disease. MASLD=metabolic dysfunction-associated steatotic liver disease. MetALD=MASLD with increased alcohol intake. MASLD-Viral=MASLD with viral hepatitis. ALD=alcohol-related steatotic liver disease. UKB= UK Biobank. NHANES=National Health and Nutrition Examination Survey.


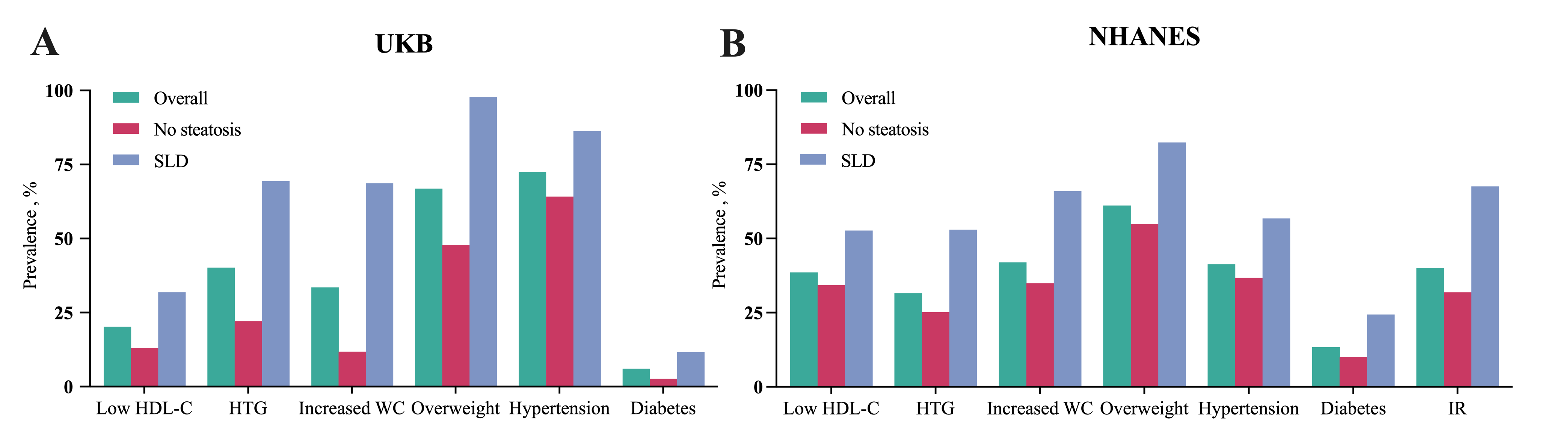


### Figure S2. The comparison of metabolic dysfunction prevalence in participants with and without SLD

(A) UKB. (B) NHANES. SLD=steatotic liver disease. Low HDL-C=low high-density lipoprotein cholesterol level. HTG=elevated triglyceride levels. WC=waist circumference. IR=insulin resistance. Specific thresholds for each metabolic dysfunction criterion are shown in the accompanying table S1. UKB=UK Biobank. NHANES=National Health and Nutrition Examination Survey.





### Figure S3. Kaplan-Meier analysis of cancer-related and all-cause mortalities in participants with NAFLD, MAFLD, and MASLD

(A) Kaplan-Meier analysis of cancer-related mortality in participants with MAFLD in the UK Biobank. (B) Kaplan-Meier analysis of cancer-related mortality in participants with MAFLD in the US NHANES. (C) Kaplan-Meier analysis of all-cause mortality in participants with MAFLD in the UK Biobank. (D) Kaplan-Meier analysis of all-cause mortality in participants with MAFLD in the US NHANES. (E) Kaplan-Meier analysis of cancer-related mortality in participants with MASLD in the UK Biobank. (F) Kaplan-Meier analysis of cancer-related mortality in participants with MASLD in the US NHANES. (G) Kaplan-Meier analysis of all-cause mortality in participants with MASLD in the UK Biobank. (H) Kaplan-Meier analysis of all-cause mortality in participants with MASLD in the US NHANES. (J) Kaplan-Meier analysis of cancer-related mortality in participants with NAFLD in the US NHANES. (I) Kaplan-Meier analysis of all-cause mortality in participants with NAFLD in the US NHANES. SLD (steatotic liver disease) serves as reference. MAFLD=metabolic dysfunction-associated fatty liver disease. MASLD=metabolic dysfunction-associated steatotic liver disease. NAFLD=non-alcoholic fatty liver disease. NHANES= National Health and Nutrition Examination Survey.

### Table S1. Detailed baseline characteristics of study participants with MAFLD

|  | **UK Biobank** | | |  | **US NHANES** | | |
| --- | --- | --- | --- | --- | --- | --- | --- |
|  | **Non-MAFLD** | **MAFLD** | ***p*-value** |  | **Non-MAFLD** | **MAFLD** | ***p*-value** |
|  | **(N = 287,450)** | **(N = 177,106)** |  |  | **(N = 10,720)** | **(N = 2,806)** |  |
| **Male, n (%)** | 99,858 (34.7) | 112,512 (63.5) | <0.001 |  | 4,984 (46.5) | 1,458 (52.0) | <0.001 |
| **Age, mean (SD) years** | 56.1 (8.2) | 57.3 (7.8) | <0.001 |  | 42.5 (15.9) | 49.3 (14.8) | <0.001 |
| **Race, n (%)** |  |  | 0.286 |  |  |  | <0.001 |
| White | 272,226 (94.7) | 16,7597 (94.6) |  |  | 6,843 (63.8) | 2,086 (74.3) |  |
| Other | 15,224 (5.3) | 9,509 (5.4) |  |  | 3,877 (36.2) | 720 (25.7) |  |
| **Alcohol intake, n (%)** |  |  | <0.001 |  |  |  | 0.248 |
| Moderate drinking | 209,117 (72.7) | 117,682 (66.4) |  |  | 9,465 (88.3) | 2,446 (87.2) |  |
| Excessive drinking | 63,748 (22.2) | 39,863 (22.5) |  |  | 632 (5.9) | 185 (6.6) |  |
| Heavy drinking | 14,585 (5.1) | 19,561 (11.0) |  |  | 623 (5.8) | 175 (6.2) |  |
| **Cigarette smoking, n (%)** |  |  | <0.001 |  |  |  | <0.001 |
| Never smoker | 168,962 (58.8) | 85,532 (48.3) |  |  | 5,229 (48.8) | 1,299 (46.3) |  |
| Past smoker | 89,987 (31.3) | 71,281 (40.2) |  |  | 2,269 (21.2) | 875 (31.2) |  |
| Current smoker | 28,501 (9.9) | 20,293 (11.5) |  |  | 3,222 (30.1) | 632 (22.5) |  |
| **Waist circumference, mean (SD) cm** | 82.7 (8.9) | 102.6 (10.0) | <0.001 |  | 90.1 (13.5) | 104.3 (13.7) | <0.001 |
| **BMI, mean (SD) kg/m^2^** | 24.9 (2.8) | 31.5 (4.5) | <0.001 |  | 26.2 (5.3) | 31.3 (6.3) | <0.001 |
| **SBP, mean (SD) mmHg** | 80.0 (10.3) | 85.8 (10.3) | <0.001 |  | 120.1 (18.8) | 129.2 (19.1) | <0.001 |
| **DBP, mean (SD) mmHg** | 136.7 (19.7) | 144.6 (18.6) | <0.001 |  | 72.5 (11.6) | 76.9 (12.0) | <0.001 |
| **Hypertension, n (%)** | 184,481 (64.2) | 152,898 (86.3) | <0.001 |  | 3,881 (36.2) | 1,704 (60.7) | <0.001 |
| **Cardiovascular disease, n (%)** | 13,257 (4.6) | 18,063 (10.2) | <0.001 |  | 542 (5.1) | 270 (9.6) | <0.001 |
| **Diabetes stage, n (%)** |  |  | <0.001 |  |  |  | <0.001 |
| Without diabetes | 216,957 (86.2) | 108,046 (67.6) |  |  | 6,461 (60.9) | 944 (34.0) |  |
| Pre-diabetes | 26,865 (10.7) | 31,284 (19.6) |  |  | 3,099 (29.2) | 1,089 (39.3) |  |
| Diabetes | 7,879 (3.1) | 20,566 (12.9) |  |  | 1,044 (9.8) | 740 (26.7) |  |
| **History of cancer, n (%)** | 29,512 (10.3) | 17,374 (9.8) | <0.001 |  | 555 (5.2) | 154 (5.5) | 0.542 |
| **Chronic viral hepatitis, n (%)** | 481 (0.2) | 213 (0.1) | <0.001 |  | 323 (3.0) | 56 (2.0) | 0.004 |
| **Fib-4, median (IQR)** | 0.8 (0.6, 1.1) | 1.2 (0.9, 1.6) | <0.001 |  | 0.7 (0.5, 1.1) | 0.9 (0.6, 1.3) | <0.001 |
| **Glycated hemoglobin, median (IQR) %** | 5.3 (5.1, 5.5) | 5.5 (5.2, 5.8) | <0.001 |  | 5.3 (5.0, 5.6) | 5.6 (5.2, 6.1) | <0.001 |
| **Total cholesterol, mean (SD) mg/dL** | 219.7 (42.1) | 220.8 (47.4) | <0.001 |  | 201.2 (42.9) | 215.6 (46.8) | <0.001 |
| **HDL-cholesterol, mean (SD) mg/dL** | 60.7 (14.7) | 48.4 (11.4) | <0.001 |  | 52.3 (15.4) | 45.1 (14.2) | <0.001 |
| **Triglycerides, median (IQR) mg/dL** | 106.8 (80.3, 144.2) | 189.0 (139.4, 258.6) | <0.001 |  | 102.0 (73.8, 150.0) | 167.0 (114.0, 248.0) | <0.001 |
| **AST, median (IQR) U/L** | 17.6 (14.0, 22.5) | 26.1 (19.9, 35.2) | <0.001 |  | 19.0 (16.0, 23.0) | 22.0 (18.0, 29.0) | <0.001 |
| **ALT, median (IQR) U/L** | 23.4 (20.3, 27.2) | 26.3 (22.4, 31.6) | <0.001 |  | 14.0 (10.0, 19.0) | 19.0 (14.0, 30.0) | <0.001 |
| **GGT, median (IQR) U/L** | 21.2 (16.2, 29.3) | 39.5 (27.9, 60.7) | <0.001 |  | 20.0 (15.0, 32.0) | 31.0 (22.0, 50.0) | <0.001 |
| **CRP, median (IQR) mg/L** | 1.0 (0.5, 1.9) | 2.1 (1.1, 4.1) | <0.001 |  | 2.1 (2.1, 3.3) | 2.1 (2.1, 6.6) | <0.001 |
| **HOMA-IR, median (IQR)** | .. | .. | .. |  | 1.8 (1.3, 2.8) | 3.7 (2.4, 6.2) | <0.001 |

Continuous data are presented as mean (standard deviation) if normally distributed or as median (interquartile range) if non-normally distributed. Categorical data are expressed as number (%). BMI=body mass index. SBP=systolic blood pressure. DBP=diastolic blood pressure. Fib-4=Fibrosis-4 index. HDL=high-density lipoprotein. AST=aspartate aminotransferase. ALT=alanine aminotransferase. GGT=gamma-glutamyl transpeptidase. CRP=C-reactive protein. HOMA-IR=homeostatic model assessment of Insulin resistance. SD=standard deviation. IQR=interquartile range. MAFLD=metabolic dysfunction-associated fatty liver disease.

### Table S2. Detailed baseline characteristics of study participants with MASLD

|  | **UK Biobank** | | |  | **NHANES** | | |
| --- | --- | --- | --- | --- | --- | --- | --- |
|  | **Non-MASLD** | **MASLD** | ***p*-value** |  | **Non-MASLD** | **MASLD** | ***p*-value** |
|  | **(N = 346,812)** | **(N = 117,744)** |  |  | **(N = 11,045)** | **(N = 2,481)** |  |
| **Male, n (%)** | 146,487 (42.2) | 65,883 (56.0) | <0.001 |  | 5,235 (47.4) | 1,207 (48.6) | 0.268 |
| **Age, mean (SD) years** | 56.2 (8.1) | 57.4 (7.9) | <0.001 |  | 42.6 (15.9) | 49.4 (15.1) | <0.001 |
| **Race, n (%)** |  |  | <0.001 |  |  |  | <0.001 |
| White | 330,432 (95.3) | 109,391 (92.9) |  |  | 7,085 (64.1) | 1,844 (74.3) |  |
| Other | 16,380 (4.7) | 8,353 (7.1) |  |  | 3,960 (35.9) | 637 (25.7) |  |
| **Alcohol intake, n (%)** |  |  | <0.001 |  |  |  | <0.001 |
| Moderate drinking | 209,055 (60.3) | 117,744 (100.0) |  |  | 9,430 (85.4) | 2,481 (100.0) |  |
| Excessive drinking | 103,611 (29.9) | 0 (0) |  |  | 817 (7.4) | 0 (0) |  |
| Heavy drinking | 34,146 (9.8) | 0 (0) |  |  | 798 (7.2) | 0 (0) |  |
| **Cigarette smoking, n (%)** |  |  | <0.001 |  |  |  | <0.001 |
| Never smoker | 190,594 (55.0) | 63,900 (54.3) |  |  | 5,312 (48.1) | 1,216 (49.0) |  |
| Past smoker | 119,040 (34.3) | 42,228 (35.9) |  |  | 2,373 (21.5) | 771 (31.1) |  |
| Current smoker | 37,178 (10.7) | 11,616 (9.9) |  |  | 3,360 (30.4) | 494 (19.9) |  |
| **Waist circumference, mean (SD) cm** | 86.0 (11.7) | 102.6 (10.3) | <0.001 |  | 90.7 (13.7) | 103.5 (14.5) | <0.001 |
| **BMI, mean (SD) kg/m^2^** | 25.9 (3.7) | 31.9 (4.7) | <0.001 |  | 26.4 (5.4) | 31.1 (6.5) | <0.001 |
| **SBP, mean (SD) mmHg** | 81.2 (10.6) | 85.1 (10.3) | <0.001 |  | 120.6 (19.0) | 128.1 (19.0) | <0.001 |
| **DBP, mean (SD) mmHg** | 138.5 (19.8) | 143.5 (18.6) | <0.001 |  | 72.8 (11.7) | 76.1 (11.9) | <0.001 |
| **Hypertension, n (%)** | 237,421 (68.5) | 99,958 (84.9) | <0.001 |  | 4,144 (37.5) | 1,441 (58.1) | <0.001 |
| **Cardiovascular disease, n (%)** | 195,162 (56.3) | 115,596 (98.2) | <0.001 |  | 572 (5.2) | 240 (9.7) | <0.001 |
| **Diabetes stage, n (%)** |  |  | <0.001 |  |  |  | <0.001 |
| Without diabetes | 25,6459 (84.1) | 68,544 (64.3) |  |  | 6,554 (60.0) | 851 (34.7) |  |
| Pre-diabetes | 35,748 (11.7) | 22,401 (21.0) |  |  | 3,237 (29.6) | 951 (38.8) |  |
| Diabetes | 12,839 (4.2) | 15,606 (14.6) |  |  | 1,134 (10.4) | 650 (26.5) |  |
| **History of cancer, n (%)** | 34,794 (10.0) | 12,092 (10.3) | 0.020 |  | 567 (5.1) | 142 (5.7) | <0.001 |
| **Chronic viral hepatitis, n (%)** | 694 (0.2) | 0 (0) | <0.001 |  | 379 (3.4) | 0 (0) | <0.001 |
| **Fib-4, median (IQR)** | 0.9 (0.7, 1.2) | 1.2 (0.9, 1.6) | <0.001 |  | 0.8 (0.5, 1.1) | 0.9 (0.6, 1.3) | <0.001 |
| **Glycated hemoglobin, median (IQR) %** | 5.3 (5.1, 5.6) | 5.5 (5.3, 5.8) | <0.001 |  | 5.3 (5.0, 5.6) | 5.6 (5.2, 6.1) | <0.001 |
| **Total cholesterol, mean (SD) mg/dL** | 220.7 (42.9) | 218.5 (47.9) | <0.001 |  | 201.9 (43.3) | 214.5 (46.2) | <0.001 |
| **HDL-cholesterol, mean (SD) mg/dL** | 59.1 (14.7) | 47.0 (10.9) | <0.001 |  | 52.2 (15.5) | 44.6 (13.7) | <0.001 |
| **Triglycerides, median (IQR) mg/dL** | 115.9 (84.8, 163.2) | 188.7 (139.4, 257.0) | <0.001 |  | 104.0 (74.0, 153.0) | 165.0 (112.0, 244.0) | <0.001 |
| **AST, median (IQR) U/L** | 18.7 (14.6, 24.9) | 25.2 (19.2, 34.1) | <0.001 |  | 19.0 (16.0, 24.0) | 21.0 (17.0, 27.0) | <0.001 |
| **ALT, median (IQR) U/L** | 24.0 (20.7, 28.1) | 25.8 (22.0, 30.8) | <0.001 |  | 14.0 (10.0, 19.0) | 19.0 (13.0, 28.0) | <0.001 |
| **GGT, median (IQR) U/L** | 23.4 (17.1, 35.7) | 35.8 (25.9, 53.5) | <0.001 |  | 21.0 (15.0, 33.0) | 29.0 (20.0, 44.0) | <0.001 |
| **CRP, median (IQR) mg/L** | 1.1 (0.6, 2.2) | 2.3 (1.2, 4.4) | <0.001 |  | 2.1 (2.1, 3.3) | 2.1 (2.1, 6.0) | <0.001 |
| **HOMA-IR, median (IQR)** | .. | .. | .. |  | 1.9 (1.3, 3.0) | 3.6 (2.3, 6.1) | <0.001 |

Continuous data are presented as mean (standard deviation) if normally distributed or as median (interquartile range) if non-normally distributed. Categorical data are expressed as number (%). BMI=body mass index. SBP=systolic blood pressure. DBP=diastolic blood pressure. Fib-4=Fibrosis-4 index. HDL=high-density lipoprotein. AST=aspartate aminotransferase. ALT=alanine aminotransferase. GGT=gamma-glutamyl transpeptidase. CRP=C-reactive protein. HOMA-IR=homeostatic model assessment of Insulin resistance. SD=standard deviation. IQR=interquartile range. MASLD=metabolic dysfunction-associated steatotic liver disease.

### Table S3. Stratified analysis of the relationship between MAFLD and mortality in the UKB and NHANES.

| Subgroup | UKB | | | |  | NHANES | | | |
| --- | --- | --- | --- | --- | --- | --- | --- | --- | --- |
|  | Total | Case | aHR (95%CI) | *P* for interaction |  | Total | Case | aHR (95%CI) | *P* for interaction |
| Overall | 463977 |  | 1.29 (1.25, 1.33) |  |  | 13271 |  |  |  |
| **Cancer-related mortality** |  |  |  |  |  |  |  |  |  |
| Sex |  |  |  | 0.001 |  |  |  |  | 0.141 |
| Male | 211832 | 9818 | 1.22 (1.17, 1.27) |  |  | 6322 | 624 | 1.19 (0.99, 1.42) |  |
| Female | 252145 | 8503 | 1.38 (1.31, 1.44) |  |  | 6949 | 525 | 1.51 (1.23, 1.84) |  |
| Race |  |  |  | 0.752 |  |  |  |  | 0.517 |
| White | 439280 | 17782 | 1.29 (1.25, 1.33) |  |  | 8779 | 771 | 1.36 (1.16, 1.60) |  |
| Other | 24697 | 539 | 1.31 (1.10, 1.56) |  |  | 4492 | 378 | 1.18 (0.90, 1.53) |  |
| Age |  |  |  | 0.005 |  |  |  |  | 0.154 |
| ＜65 | 395698 | 12657 | 1.33 (1.28, 1.38) |  |  | 11268 | 799 | 1.36 (1.15, 1.61) |  |
| ≥65 | 68279 | 5664 | 1.22 (1.15, 1.29) |  |  | 2003 | 350 | 1.10 (0.86, 1.39) |  |
| Smoke |  |  |  | <0.001 |  |  |  |  | 0.240 |
| Never | 254205 | 7275 | 1.37 (1.31, 1.44) |  |  | 6406 | 350 | 1.57 (1.24, 1.98) |  |
| Previous | 161077 | 7722 | 1.32 (1.26, 1.39) |  |  | 3106 | 360 | 1.22 (0.97, 1.52) |  |
| Current | 48695 | 3324 | 1.06 (0.98, 1.14) |  |  | 3759 | 439 | 1.21 (0.95, 1.55) |  |
| CVD |  |  |  | 0.050 |  |  |  |  | 0.877 |
| No | 432688 | 16109 | 1.31 (1.26, 1.35) |  |  | 12462 | 1046 | 1.30 (1.13, 1.50) |  |
| Yes | 31289 | 2212 | 1.15 (1.05, 1.26) |  |  | 809 | 103 | 1.23 (0.81, 1.87) |  |
| Cancer |  |  |  | 0.017 |  |  |  |  | 0.819 |
| No | 417132 | 13720 | 1.31 (1.27, 1.36) |  |  | 12571 | 994 | 1.29 (1.12, 1.50) |  |
| Yes | 46845 | 4601 | 1.21 (1.14, 1.29) |  |  | 700 | 155 | 1.22 (0.83, 1.78) |  |
| **All-cause mortality** |  |  |  |  |  |  |  |  |  |
| Sex |  |  |  | <0.001 |  |  |  |  | 0.004 |
| Male | 211832 | 22149 | 1.23 (1.19, 1.26) |  |  | 6322 | 2514 | 1.32 (1.21, 1.44) |  |
| Female | 252145 | 15407 | 1.52 (1.47, 1.57) |  |  | 6949 | 2284 | 1.61 (1.47, 1.77) |  |
| Race |  |  |  | 0.058 |  |  |  |  | 0.871 |
| White | 439280 | 36222 | 1.34 (1.31, 1.37) |  |  | 8779 | 3262 | 1.44 (1.34, 1.56) |  |
| Other | 24697 | 1334 | 1.48 (1.33, 1.66) |  |  | 4492 | 1536 | 1.42 (1.25, 1.60) |  |
| Age |  |  |  | <0.001 |  |  |  |  | <0.001 |
| ＜65 | 395698 | 24734 | 1.41 (1.37, 1.44) |  |  | 11268 | 2959 | 1.66 (1.53, 1.80) |  |
| ≥65 | 68279 | 12822 | 1.24 (1.20, 1.29) |  |  | 2003 | 1839 | 1.07 (0.96, 1.18) |  |
| Smoke |  |  |  | <0.001 |  |  |  |  | 0.001 |
| Never | 254205 | 14545 | 1.46 (1.41, 1.51) |  |  | 6406 | 1764 | 1.59 (1.44, 1.77) |  |
| Previous | 161077 | 15954 | 1.40 (1.35, 1.45) |  |  | 3106 | 1531 | 1.22 (1.09, 1.36) |  |
| Current | 48695 | 7057 | 1.05 (1.00, 1.10) |  |  | 3759 | 1503 | 1.56 (1.38, 1.77) |  |
| CVD |  |  |  | 0.001 |  |  |  |  | 0.216 |
| No | 432688 | 30861 | 1.36 (1.33, 1.40) |  |  | 12462 | 4125 | 1.45 (1.35, 1.56) |  |
| Yes | 31289 | 6695 | 1.23 (1.17, 1.29) |  |  | 809 | 673 | 1.18 (1.00, 1.39) |  |
| Cancer |  |  |  | <0.001 |  |  |  |  | 0.460 |
| No | 417132 | 30319 | 1.36 (1.33, 1.39) |  |  | 12571 | 4317 | 1.43 (1.33, 1.53) |  |
| Yes | 46845 | 7237 | 1.25 (1.19, 1.31) |  |  | 700 | 481 | 1.29 (1.04, 1.60) |  |

Results were obtained with Cox proportional hazards. Models were adjusted for age (category), sex, race,cigarette smoking, alcohol intake, Fib-4, CVD, history of cancer, and viral hepatitis. aHR=adjusted hazard ratio. CI=confidence interval. CVD=cardiovascular disease. Fib-4=Fibrosis-4 index. MAFLD=metabolic dysfunction-associated fatty liver disease. UKB=UK Biobank. NHANES=National Health and Nutrition Examination Survey.

### Table S4. Definition factors of MAFLD and MASLD

|  | **MAFLD** | **MASLD** |
| --- | --- | --- |
| **Definition** | MAFLD was defined as the presence of SLD and the presence of one of the following three conditions (overweight/obese, type 2 diabetes mellitus or metabolic abnormalities). | MASLD was defined as the presence of SLD and one or more of the following cardiometabolic risk factors (overweight/obesity, prediabetes/type 2 diabetes mellitus, hypertension, hypertriglyceridemia or low HDL cholesterol). |
| **Steatotic Liver Disease** | UK Biobank: fatty liver index ≥ 60; NHANES: steatotic liver disease was diagnosed by having the presence of moderate or severe hepatic steatosis. | UK Biobank: fatty liver index ≥ 60; NHANES: steatotic liver disease was diagnosed by having the presence of moderate or severe hepatic steatosis. |
| **Alcohol drinking** |  | UK Biobank: alcohol consumption ≤ 20 g/day for women and ≤ 30 g/day for men; NHANES: alcohol consumption ≤ 2 drinks/day for males and ≤ 1 drink/day for females. |
| **Overweight or obesity** | NHANES and UK Biobank: BMI ≥ 25 kg/m^2^. | NHANES and UK Biobank: BMI ≥ 25 kg/m^2^ or WC ≥ 94 cm for males or ≥ 80 cm for females. |
| **Type 2 diabetes mellitus** | UK Biobank: serum glucose level ≥ 11.1 mmol/L (200 mg/dl); HbA1c ≥ 48 mmol/mol (6.5%) or type 2 diabetes or regularly took medication for diabetes. NHANES: HbA1c ≥ 48 mmol/mol (6.5%) or fasting serum glucose ≥ 7.0 mmol/L (≥126 mg/dL) or 2-hour post-load glucose levels ≥ 11.1 mmol/L (≥ 200 mg/dL) or type 2 diabetes or or regularly took medication for diabetes. |  |
| **Metabolic abnormalities** | UK Biobank and NHANES: presence of at least two metabolic abnormalities. 1) Including increased WC (≥ 102 cm for males and ≥88 cm for females ); 2) Hypertension (systolic ≥130 or diastolic ≥85 mmHg or use of antihypertensive medication); 3) Hypertriglyceridemia (triglyceridemia ≥ 1.7 mmol/L [150 mg/dL] or use of a relevant drug); 4) Low HDL cholesterol (HDL-cholesterol ≤ 1.0 mmol/L [40 mg/dL] for males and ≤ 1.3 mmol/L [50 mg/dL] for females or use of a relevant drug); 5) Prediabetes (HbA1c 39-47 mmol/mol); 6) Subclinical inflammation (C-reactive protein > 2 mg/L); 7) Insulin resistance: HOMA-IR values >2.5 (insulin resistance not applicable in the UK Biobank). |  |
| **Prediabetes or type 2 diabetes mellitus** |  | UK Biobank: HbA1c ≥ 5.7% (39 mmol/L) or or serum glucose level ≥11.1 mmol/L (200 mg/dl) or type 2 diabetes or regularly took medication for diabetes; NHANES: HbA1c ≥ 5.7% (39 mmol/L) or fasting serum glucose ≥ 5.6 mmol/L ( ≥ 100 mg/dL) or 2-hour post-load glucose levels ≥ 7.8 mmol/L (≥140 mg/dL) or type 2 diabetes or regularly took medication for diabetes. |
| **Hypertension** |  | UK Biobank and NHANES: blood pressure ≥ 130/85 mmHg or specific antihypertensive drug treatment |
| **Hypertriglyceridemia** |  | UK Biobank and NHANES: triglycerides ≥ 1.70 mmolL [150 mg/dL] OR lipid lowering treatment. |
| **Low HDL cholesterol** |  | UK Biobank and NHANES: HDL-cholesterol ≤ 1.0 mmol/L [40 mg/dL] for males and ≤ 1.3 mmol/L [50 mg/dL] for females or use of a relevant drug. |

MAFLD=metabolic dysfunction-associated fatty liver disease. MASLD=metabolic dysfunction-associated steatotic liver disease. NHANES=US National Health and Nutrition Examination Survey. SLD=steatotic liver disease. BMI=body mass index. BP=blood pressure. HbA1c=glycosylated hemoglobin. HDL=high-density lipoproteins. HOMA-IR: homeostasis model assessment-insulin resistance. WC=waist circumference.

### Table S5. Codes used to identify medications in the UKB cohort

|  | **Coding** |
| --- | --- |
| **Hypolipidemic drugs** |  |
| HDL-raising drugs | - 1140910670 niacin - 1140861868 nicotinic acid product - 1140861954 fenofibrate - 1141182910 fenogal 200mg capsule - 1141157262 gemfibrozil product - 1140861856 gemfibrozil |
| Triglyceride lowering drugs | - 1140861868 nicotinic acid product - 1140910670 niacin - 1141157260 bezafibrate product - 1140861924 bezafibrate - 1140861954 fenofibrate - 1141157262 gemfibrozil product  - 1140861856 gemfibrozil - 1140862026 ciprofibrate - 1140861944 clofibrate - 1141168568 bezagen xl 400mg m/r tablet - 1140861926 bezalip 200mg tablet - 1140861928 bezalip-mono 400mg m/r tablet - 1141182910 fenogal 200mg capsule - 1141201306 fibrazate xl 400mg m/r tablet - 1141162544 lipantil micro 67mg capsule - 1141175908 liparol xl 400mg m/r tablet - 1140861858 lopid 300 capsule - 1140862028 modalim 100mg tablet - 1141172214 supralip 160mg m/r tablet - 1141171548 zimbacol xl 400mg m/r tablet |
| Statins | - 1141146234 atorvastatin - 1141192410 rosuvastatin - 1140861958 simvastatin - 1141192414 crestor 10mg tablet - 1140881748 zocor 10mg tablet - 1141200040 zocor heart-pro 10mg tablet - 1141146138 lipitor 10mg tablet - 1140864592 lescol 20mg capsule |
| **Glucose lowering drugs** |  |
| Metformin | - 1140884600 metformin - 1140874686 glucophage 500mg tablet  - 1140921964 glucamet 500 tablet - 1140874690 orabet 500mg tablet |
| Sulfonylureas | - 1140874646 glipizide - 1141157284 glipizide product - 1140874744 gliclazide - 1141152590 glimepiride - 1140874658 gliquidone - 1140874666 tolanase 100mg tablet - 1140874664 tolazamide - 1140874650 glibenese 5mg tablet - 1140874652 minodiab 2.5mg tablet - 1140874674 tolbutamide - 1140874678 glyconon 500mg tablet - 1140874706 chlorpropamide - 1140874660 glurenorm 30mg tablet - 1140874726 semi-daonil 2.5mg tablet  - 1140874728 euglucon 2.5mg tablet  - 1140874746 diamicron 80mg tablet - 1140874680 rastinon 500mg tablet - 1141156984 amaryl 1mg tablet - 1140874718 glibenclamide - 1140874724 daonil 5mg tablet - 1140874736 diabetamide 2.5mg tablet - 1140874712 diabinese 100mg tablet - 1140874740 calabren 2.5mg tablet - 1141169504 diaglyk 80mg tablet - 1140857590 libanil 2.5mg tablet - 1140874732 malix 2.5mg tablet |
| Thiazolidinediones | - 1141171646 pioglitazone - 1141177600 rosiglitazone - 1141171652 actos 15mg tablet - 1141153254 troglitazone  - 1141177606 avandia 4mg tablet |
| Metformin and rosiglitazone | - 1141189094 avandamet 1mg / 500mg tablet - 1141189090 rosiglitazone 1mg / metformin 500mg tablet |
| Alpha-glucosidase inhibitors | - 1140868902 acarbose  - 1140868908 glucobay 50mg tablet |
| Meglitinides | - 1141173786 starlix 60mg tablet - 1141173882 nateglinide - 1141168660 repaglinide - 1141168668 novonorm 0.5mg tablet |
| Insulin | - 1140883066 insulin product |
| **Aspirin** | - 1140909480 aspro clear maximum strength soluble tablet - 1140861806 aspirin 75mg tablet - 1140864860 nu-seals aspirin 75mg e/c tablet - 1140868226 aspirin - 1140868282 aspirin+methocarbamol 325mg/400mg tablet - 1140872040 aspirin+metoclopramide 325mg/5mg effervescent tablet - 1140882108 aspirin+cyclizine hydrochloride 500mg/25mg tablet - 1140882190 aspirin+glycine 500mg/133mg dispersible tablet - 1140882268 aspirin+codeine 300mg/8mg tablet - 1140882392 aspirin+codeine - 1141163138 aspirin+papaveretum 500mg/7.71mg dispersible tablet - 1141164044 isosorbide mononitrate+aspirin - 1141167844 dipyridamole+aspirin - 1140861808 disprin cv 100mg m/r tablet - 1140882192 disprin direct dispersible table - 1140861804 angettes 75mg table - 1141167848 asasantin retard m/r capsule - 1140917114 alka-seltzer tablet - 1140856310 aspergum 227mg chewing gum - 1140925942 caprin 75mg e/c tablet - 1141167026 caspac xl 162.5mg m/r capsule - 1140856312 claradin 300mg tablet - 1141151924 enprin 75mg e/c tablet - 1141164050 imazin xl 60mg/75mg m/r tablet - 1140863514 laboprin dl 900mg sachet - 1140856314 laboprin 300mg tablet - 1141177826 micropirin 75mg e/c tablet - 1140856212 paynocil 600mg tablet - 1140861800 platet 100mg effervescent tablet - 1140917408 postmi 75mg dispersible tablet - 1140856214 solprin 300mg dispersible tablet - 1141167848 asasantin retard m/r capsule |

### Table S6. ICD-10 and ICD-9 codes used to determine liver disease and medical history

|  | **ICD-10 codes** | **ICD-9 codes** |
| --- | --- | --- |
| Liver cancer | C22 | 155.0-155.3 |
| Liver cirrhosis | K70.2, K70.3, K71.7, K74.1, K74.2, K74.4, K74.5, K74.6, K76.6 | 571.2, 571.5, 571.6 |
| Chronic viral hepatitis | B16-B18 | 0703 |
| Type 2 diabetes mellitus | E11 | 250 |
| Cancer | C00-C97 | 140-208 |
| Hypertension | I10-I13, I15, O10 | 401-405 |
| CVD | I20-25, I60-64 | 410-414, 430-434, 436 |

ICD=International Classification of Diseases. CVD=cardiovascular disease.

### Table S7. Calculation of pure alcohol intake in the UKB cohort

|  | **Unit** | **Pure alcohol (g/100 ml)** | **Intake frequency** | **Pure alcohol intake (g/day)** |
| --- | --- | --- | --- | --- |
| **Red wine** |  |  |  |  |
| Red wine | Glass (125 ml/glass) | 13 | - Special occasion only  - 1-3 times/month | (red wine×13/100×125)/30 |
| Red wine | Glass (125 ml/glass) | 13 | - 1-2 times/week  - 3-4 times/week  - Daily or almost daily | (red wine×13/100×125)/7 |
| **White wine** |  |  |  |  |
| White wine/champagne | Glass (125 ml/glass) | 12 | - Special occasion only  - 1-3 times/month | (white wine×12/100×125)/30 |
| White wine/champagne | Glass (125 ml/glass) | 12 | - 1-2 times/week  - 3-4 times/week  - Daily or almost daily | (white wine×12/100×125)/7 |
| **Beer/cider** |  |  |  |  |
| Beer, bitter, lager, stout, ale, guinness | Pint (568.3 ml/pint) | 4.5 | - 1-2 times/week  - 3-4 times/week  - Daily or almost daily | (beer×4.5/100×568.3)/30 |
| Beer, bitter, lager, stout, ale, guinness | Pint (568.3 ml/pint) | 4.5 | - Special occasion only  - 1-3 times/month | (beer×4.5/100×568.3)/7 |
| **Spirits** |  |  |  |  |
| Whisky, gin, rum, vodka, brandy | Measure (25 ml/measure) | 40 | - 1-2 times/week  - 3-4 times/week  - Daily or almost daily | (spirits×40/100×25)/30 |
| Whisky, gin, rum, vodka, brandy | Measure (25 ml/measure) | 40 | - Special occasion only  - 1-3 times/month | (spirits×40/100×25)/7 |
| **Fortified wine** |  |  |  |  |
| Sherry, port, vermouth | Glass (125 ml/glass) | 18 | - 1-2 times/week  - 3-4 times/week  - Daily or almost daily | (fortified wine×18/100×75)/30 |
| Sherry, port, vermouth | Glass (125 ml/glass) | 18 | - Special occasion only  - 1-3 times/month | (fortified wine×18/100×75)/7 |
| **Others** |  |  |  |  |
| Other alcoholic drinks | Glass (125 ml/glass) | 4 | - 1-2 times/week  - 3-4 times/week  - Daily or almost daily | (other wine×4/100×275)/30 |
| Other alcoholic drinks | Glass (125 ml/glass) | 4 | - Special occasion only  - 1-3 times/month | (other wine×4/100×275)/7 |

During a self-completed touchscreen interview taken at baseline appointment, participants were asked about their current drinking status (never, previous, current, prefer not to say) and were asked to report their average weekly and monthly alcohol consumption of a range of drink types (red wine, white wine/champagne, spirits, beer/cider, fortified wine, other wine). From these measures we derived an average intake of alcohol consumption in gram per day.
